# CD95L Inhibition Impacts Gemcitabine-Mediated Effects and Non-Apoptotic Signaling of TNF-α and TRAIL in Pancreatic Tumor Cells

**DOI:** 10.3390/cancers13215458

**Published:** 2021-10-30

**Authors:** Khalid Rashid, Christian Röder, Freya Goumas, Jan-Hendrik Egberts, Holger Kalthoff

**Affiliations:** 1 Institute for Experimental Cancer Research, University Medical Centre Schleswig-Holstein (UKSH), Campus Kiel, 24105 Kiel, Germany; rashidk@fudan.edu.cn (K.R.); c.roeder@email.uni-kiel.de (C.R.); 2 Department of General, Visceral-, Thoracic-, Transplantation- and Paediatric Surgery, University Medical Centre Schleswig-Holstein (UKSH), Campus Kiel, 24105 Kiel, Germany; agoumas@web.de (F.G.); j.egberts@ik-h.de (J.-H.E.); 3 Department of Visceral Surgery, Israelitisches Krankenhaus, 22297 Hamburg, Germany

**Keywords:** gemcitabine, death ligands, chemoresistance, PDAC, metastasis

## Abstract

**Simple Summary:**

Death receptors may induce cellular death upon ligand binding but also activate other pro-inflammatory mechanisms in cancer cells, which may increase malignancy. The transcription factor NFkB works as a central hub in this system and connects the signaling of three major death ligands: TNF, CD95L and TRAIL. In this study, we analyzed the impact of a recombinant inhibitor of CD95L on the respective tri-lateral cross-talk using highly malignant pancreatic cancer cells as a model and in context of the standard chemotherapeutic agent, gemcitabine. We show that the specific “uni-lateral” inhibitor also clearly impacts the two other cytokines and importantly reduces the pro-inflammatory status of pancreatic cancer cells as well as their stem cell phenotype. Consequently, the local recurrent tumor growth after surgery of the primary tumor was reduced. Further, the unwanted, pro-inflammatory side-effects of gemcitabine are inhibited.

**Abstract:**

Despite the potential apoptotic functions, the CD95/CD95L system can stimulate survival as well as pro-inflammatory signaling, particularly through the activation of NFκB. This holds true for the TNF/TNFR and the TRAIL/TRAILR systems. Thus, signaling pathways of these three death ligands converge, yet the specific impact of the CD95/CD95L system in this crosstalk has not been well studied. In this study, we show that gemcitabine stimulates the expression of pro-inflammatory cytokines, such as IL6 and IL8, under the influence of the CD95/CD95L system and the pharmacological inhibitor, sCD95Fc, substantially reduced the expression in two PDAC cell lines, PancTuI-luc and A818-4. The stem cell phenotype was reduced when induced upon gemcitabine as well by sCD95Fc. Moreover, TNF-α as well as TRAIL up-regulate the expression of CD95 and CD95L in both cell lines. Conversely, we detected a significant inhibitory effect of sCD95Fc on the expression of both IL8 and IL6 induced upon TNF-α and TRAIL stimulation. In vivo, CD95L inhibition reduced xeno-transplanted recurrent PDAC growth. Thus, our findings indicate that inhibition of CD95 signaling altered the chemotherapeutic effects of gemcitabine, not only by suppressing the pro-inflammatory responses that arose from the CD95L-positive tumor cells but also from the TNF-α and TRAIL signaling in a bi-lateral crosstalk manner.

**Highlights:**

(1) The CD95/CD95L system plays an important role in non-apoptotic signaling;
(2) The CD95/CD95L system influences TNF-α/TRAIL-induced pro-inflammatory signaling;
(3) The blockade of CD95L may enhance the chemotherapeutic effects of gemcitabine by lowering the drug-induced pro-inflammatory signaling;
(4) The respective inhibitor sCD95Fc reduces in vivo pancreatic tumor growth in an adjuvant setting.

## 1. Introduction

Pancreatic ductal adenocarcinoma (PDAC) is one of the deadliest and highly therapy-resistant types of cancer amongst all malignancies and has the poorest prognosis. PDAC causes thousands of deaths every year, with an overall five-year survival rate of 5–7% in Western countries [1,2]. Palliative chemotherapy is the only option for the treatment of advanced PDAC due to the major complexity of late symptoms and the inaccessible location of the pancreas for complete resection of locally and/or distant advanced tumors [3]. Gemcitabine is the most widely used chemotherapeutic agent for the treatment of PDAC. However, it exhibits few benefits in terms of tumor response and prolongation of patient survival due to the problem of chemoresistance in PDAC [4].

It has previously been well-described that most of the clinically used chemotherapeutic drugs, including gemcitabine, stimulated enhanced surface protein levels of CD95 and CD95L, the latter a member of the death receptor ligand (DRL) superfamily of TNFSF, which also includes TNF-α and TRAIL. This elevated surface expression of CD95 and CD95L could be either NFκB or AP-1 or MAPK dependent [5,6]. However, even after enhanced surface expression of CD95 and CD95L, most of the tumor cells show resistance towards CD95 mediated apoptosis especially after chemotherapy [7]. It has been also reported that gemcitabine not only selects the resistant tumor cells but also shows some differentiating effects on PDAC towards enhanced stem cell phenotype [8].

In addition, it has been demonstrated that an increase in pro-inflammatory cytokines (PICs) as well as tumor-associated proteases (TAPs) is associated with poor prognosis, which protects PDAC cells from the chemotherapy-induced apoptotic cell death [9,10]. An elevated level of TNF-α and IL6 has been found in the serum of cancer patients treated with gemcitabine [11]. The potential for individual PICs to decrease chemotherapeutic efficacy indicates that they may be candidates for testing anti-inflammatory therapy in advanced PDAC patients.

Apoptotic resistance to chemotherapy becomes a universal problem and a major cause of treatment failure in PDAC [12,13]. Besides the enormous involvement in apoptotic functions [14], the CD95/CD95L system has a major impact on various non-apoptotic processes (due to tumor cell resistance) through the activation of various pro-inflammatory as well as pro-growth signaling cascades, including NFκB [15,16,17,18,19,20,21]. In previous studies, it was also uncovered that the CD95/CD95L system is involved in the enhancement of stem cell phenotype (CD133 marker) of tumor cells, contributing to the development of an enhanced metastatic phenotype with high chemo-resistant properties [22].

Similar to CD95L, TNF-α and/or TRAIL are also expressed in tumor cells of different origins, including PDAC. These “death ligands” may actively promote tumor cell growth, migration, angiogenesis, and metastasis [23,24,25,26]. Further, TNF-α or TRAIL signaling also play important roles in the induction of PICs as well as TAPs as has been reported for pancreatic tumor cells in an NFκB-dependent manner [27,28]. Since NFκB is one of the predominant factors that is not only involved in the induction of various potent inflammatory molecules (IL8, IL6, MMPs, u-PA, TNF-α), it also causes major hindrance in the apoptotic pathways mediated by these DRLs [29,30]; this hindrance might play a pivotal role in the chemoresistance against gemcitabine through the induction of pro-inflammatory pathways activated by the death ligands: TNF-α, TRAIL and CD95L.

In agreement with all these findings, we hypothesized that these three major death ligands TNF-α, TRAIL and CD95L might play a key crosstalk role in the inflammation-associated progression of PDAC, all being connected to a prototypic pro-inflammatory transcription factor, such as NFκB, which acts as a potential molecular bridge between tumor cells and inflammatory cells [31,32].

In this study, to define the resistance mechanisms of gemcitabine and the potential crosstalk role of the closely related death ligands TNF-α and/or TRAIL with CD95L in a bi-lateral manner, we investigated the role of the CD95/CD95L system in contributing to gemcitabine resistance in the context of TNF-α- and TRAIL-driven pro-inflammatory signaling. We also analyzed the effect of CD95L inhibition on the gemcitabine-induced death of PDAC cells for an improved therapeutic index of the drug under the influence of reduced pro-inflammatory responses mediated by the death ligands TNF-α, TRAIL and CD95L. Finally, the in vivo effects of sCD95Fc were shown in an experimental xenotransplant model closely mimicking adjuvant therapy after primary orthotopic tumor resection.

## 2. Materials and Methods

### 2.1. Cell Culture

Human pancreatic cancer cell lines (A818-6, A818-4, Colo-357, Panc89 and PancTuI-luc) and their suppliers have been described previously [33]. The human T-cell line Jurkat overexpressing CD95L (JFL.13.3 cell line) and the CD95L-negative breast cancer cell line MDA-MB-468 were obtained from the Institute of Immunology, Kiel University, Germany and ATCC, USA respectively. All pancreatic cancer cell lines as well as JFL.13.3 and MDA-MB-468 were grown in RPMI-1640 medium supplemented with 10% FCS, 2 mM glutamine, and 1 mM sodium pyruvate (all from Thermo Fisher Scientific, Darmstadt, Germany) at 37 °C in a water-saturated atmosphere containing 5% CO_2_.

### 2.2. Isolation of Total Cellular RNA

Total cellular RNA was prepared using PeqGold total RNA isolation kits (Peqlab/VWR Avantor, Darmstadt, Germany) according to the manufacturer’s protocol. Briefly, approximately 2.8 × 10^5^ cells were seeded into 6-well plates and after overnight growth, cells were treated with the following reagents in the presence or absence of CD95L inhibitor (sCD95Fc) in two different concentrations (20 and 60 μg/mL, respectively): TNF-α (Knoll/BASF, Ludwigshafen, Germany), 50 ng/mL, TRAIL (PeproTech, Hamburg, Germany), 50 ng/mL and CD95L (IBA, Göttingen, Germany), 100 ng/mL for 36 h. After incubation with these factors, cells were scraped off with RNA Lysis Buffer T (supplied with PeqGold total RNA isolation kits) and the RNA was isolated and finally dissolved in 50 μL RNAse-free water. The RNA concentration was measured by photometry using a Nanodrop^®^ ND-1000 UV spectrophotometer (Peqlab/VWR Avantor) at a wavelength of 260 nm with reference wavelengths of 230 nm and 280 nm to estimate the purity of the preparations. Additionally, RNA concentration and integrity were determined by either using the Experion RNA StdSens analysis kit in combination with an Experion microfluidics electrophoresis system following the manufacturer’s protocol (Bio-Rad, München, Germany).

### 2.3. Synthesis of Complementary DNA (cDNA)

Complementary DNA was synthesized using the Maxima First Strand cDNA kit (Thermo Fisher Scientific) in a volume of 20 µL following the manufacturer’s protocol. Briefly, 2–5 μg RNA was diluted in 14 μL RNAse-free H_2_O and mixed with 4 μL of 5× reaction mix and 2 μL of 10× Maxima enzyme mix. The mixture was incubated at 25 °C for 10 min and subsequently heated to 50 °C for 30 min and reaction was stopped by heating to 85 °C for 5 min. The cDNAs were stored frozen at −20 °C.

### 2.4. PerfectProbe Real Time PCR Assay

Quantitative real-time PCR gene expression profiling was performed using PerfectProbe Precision PCR Master Mix (PrimerDesign Limited, Southampton, UK) and run in a StepOne Plus Real-Time PCR System (Thermo Fisher Scientific). Premixed primers and a FAM dye-labeled PerfectProbe were predesigned by the manufacturer (Primer Design Limited, UK). All used probes are summarized in Supplementary Table S1.

All experiments were performed in technical duplicates for each of the three biological replicates in 96-well plates. The used fluorogenic probes were labeled at the 5′ end with FAM and at the 3′ end with an MGB (minor groove binder)-nonfluorescent quencher. Reactions were performed in a final volume of 10.5 μL containing 50 ng cDNA, 100 nM of forward and reverse primers, 200 nM fluorogenic probe (total mix of 300 nM) and 5 μL of 2× PerfectProbe Precision PCR Master Mix (PrimerDesign Limited, Southampton, UK). The fluorescent signal detection used ROX as the internal passive reference dye. The parameters of the PCR reaction were 10 min at 95 °C, 15 s at 95 °C followed by 50 cycles of 30 s at 50 °C and 15 s at 72 °C. The relative expression of each sample was calculated using the qBase software (Biogazelle, Zwijnaarde, Belgium) and the 2^−ΔΔCT^ method.

### 2.5. Cell Surface Protein Analysis by Flow Cytometry

After cell growth under the indicated conditions, adherent cells were washed with PBS, and detached using Accutase (PAA, Cölbe, Germany) for counting and staining. To analyze the surface expression of CD95 and CD95L, 2.0 × 10^5^ cells were re-suspended in 100 μL of FACS buffer (PBS with 0.05% sodium azide) and incubated with 10 μg/mL of anti-CD95L antibodies (PE-NOK1-BioLegend/BIOZOL, Eching, Germany) or anti-CD95 antibodies (PE-DX2-BioLegend) or with PE-IgG1 (BD Pharmigen, San Jose, CA, USA) antibodies as an isotype control for 45 min at 4 °C in dark.

The PancTuI-luc and A818-4 cells were washed twice with ice-cold FACS buffer, centrifuged and re-suspended in 300 μL of 1% PFA/PBS and either measured immediately or stored at 4 °C in the dark for later use. Additionally, for estimation of the number of dead cells, propidium iodide (PI) (1 μg/mL) was added prior to re-suspension into fixing solution.

The staining was analyzed by a FACSCalibur™ flow cytometer (Becton Dickinson Heidelberg, Germany) and the cell populations were gated for living cells and the staining of isotype control was compared to the surface staining with anti-CD95L and anti-CD95 antibody. 10,000 events were acquired per sample and the mean fluorescence intensity of four independent experiments was analyzed using different cytometry analysis software packages (CELLQuest, Win MDI).

We determined the percentage of positively stained cells by using the FACS software for isotype control (ISO)- and target of interest (TOI)-antibody-stained cells under both untreated as well as treated conditions represented by differently colored histograms for the respective groups in each FACS data analysis. To calculate the percentage of the positively stained cells, we subtracted the background staining (ISO) from test antibodies staining (TOI) under both untreated and treated conditions and then the final ratio as fold change obtained by dividing the corrected treated; (TOI_t_−ISO_t_) from the corrected untreated; (TOI_u_−ISO_u_).

### 2.6. WST-1 Proliferation Assay

In this experiment, the PancTuI-luc and A818-4 cells were seeded in 96-well plates at a density of 1.0 × 10^4^ or 0.7 × 10^4^, respectively, for 24 h or 48 h of treatment and grown in the standard cell culture medium. After 24 h of incubation, the old medium was replaced with fresh medium (100 μL/well) with or without an escalating amount (mentioned in result section) of gemcitabine, sCD95Fc and Remicade/infliximab or TNF-α, TRAIL and rCD95L for 24 h and 48 h. In addition, to test the sensitization effects of gemcitabine (20 μM) or TNF-α (50 ng/mL) or TRAIL (50 ng/mL) and/or rCD95L (100 ng/mL) on cells after CD95L inhibition, we pretreated the cells with sCD95Fc (60 μg/mL) prior to addition of these agents. The effect of death ligands on cell proliferation was tested by using WST-1 proliferation assay. Briefly, after an appropriate time of treatment of the different factors, WST-1 reagent (10 μL/well; Roche, Mannheim, Germany) was added to each well for 2 h of cell culture. The absorbance was measured photometrically using a Sunrise microplate reader at 460 nm (TECAN, Crailsheim, Germany).

### 2.7. Analysis of Pancreatic Tumor Cell Conditioned Media by IL8 ELISA

Pancreatic tumor cells PancTuI-luc and A818-4 were seeded in 6-well plates and grown to 80% confluence. The medium was then replaced by a minimal volume (1 mL) of normal growth medium containing either 10% FCS and treatment factors and incubation was continued for 36 h. Conditioned supernatant was collected in an Eppendorf and dead cells and debris were removed by centrifugation at 13,000 rpm for 30 min and stored immediately at −80 °C for subsequent analysis of the presence of secreted IL8 by an IL8-specific ELISA kit (R&D Systems, Wiesbaden, Germany) following the manufacturer’s protocol.

### 2.8. Laboratory Animals

Four-week-old female severe combined immunodeficient beige (SCID/bg) mice (C.B.-17/IcrHsd-Prkdc-scid/LysCrl) were obtained from Charles River Laboratories (Sulz-feld, Germany). They were housed under sterile conditions, in which bedding, food and water were autoclaved (Scantainer, Scanbur A/S, Karlslunde, Denmark) and allowed to become acclimatized for 10 days. Animal experiments and care were in accordance with the guidelines of institutional authorities and approved [V 312-72241.121-7(28-3/09)].

### 2.9. Orthotopic Xenotransplantation of Human PDAC Cells and Treatment in the Palliative Setting

The technique of tumor cell injection was performed as previously described in detail [34,35,36,37]. In brief, PancTuI-luc cells were trypsinized and suspended in Matrigel™ (BD Bioscience, Heidelberg, Germany) at a concentration of 4 × 10^7^ cells/mL and stored on ice. The operative procedures were performed in general anesthesia by injecting Midazolam/Fentanyl/Medetomidine (dosage 5.0 mg/kg/0.05 mg/kg/0.5 mg/kg bwt) intraperitoneally (i.p.). After median laparotomy, 25 µL of the tumor cell suspension containing 1 × 10^6^ cells were slowly injected into the pancreatic tail. The pancreas was replaced, and the abdominal wall closed using Vicryl 6/0 (Ethicon, Norderstedt, Germany). The recovery of the mice was carefully supervised. Buprenorphine at 0.1 mg/kg bwt was injected subcutaneously (s.c.) immediately after cell inoculation and once daily for two further postoperative days for analgesia. After acclimatization, mice were randomly assigned into the treatment groups. Treatment via i.p injection started three days after tumor cell inoculation and was repeated twice a week. All mice survived the operative procedure for the palliative treatment and were randomly assigned into five groups. Four groups received sCD95Fc in different concentrations (12.5, 25, 50 or 100 µg/g bwt). The control group received sodium chloride 0.9% (B. Braun, Melsungen, Germany). All mice were inspected daily for complications. One mouse died on the 17th day after surgery. 28 days after tumor cell injection, the mice were sacrificed. Tumors were removed and weighed. Subsequently, the tumor was cut into two equal parts, which were immediately either frozen in liquid nitrogen or formalin-fixed and later paraffin-embedded (FFPE). The mice were carefully observed for any macroscopic metastases in liver, spleen and mesenterium.

### 2.10. Adjuvant Setting: Relaparotomy, Tumor Resection and Treatment

For the adjuvant therapy, ten days after tumor cell inoculation, the tumors were resected as previously described in detail [34,35,36,37]. In this setting, four mice died in the first two days after relaparotomy, and one mouse of the control group died later due to tumor progression. Treatment of the mice started three days after tumor resection. Mice were randomly assigned into two groups. Sodium chloride 0.9% or sCD95Fc (25 µg/g bwt) were injected i.p. twice a week. Animals were sacrificed on the 26th day after relaparotomy. Organs and tumors were examined as described for the palliative setting.

### 2.11. Tissue Staining by Immunohistochemistry

#### 2.11.1. Cryo-Tissues

The 6 μm thick consecutive cryostat sections were mounted on uncovered glass slides, air-dried for at least 1 h at room temperature and stored vacuum-sealed at −20 °C. For the staining procedure, the sections were dried again for 30 min at room temperature, fixed in acetone (Merck, Darmstadt, Germany) for 10 min and air-dried again for 10 min. Then the slides were washed in phosphate-buffered saline (PBS) and incubated with 4% bovine serum albumin in PBS (Biomol, Hamburg, Germany) for 20 min followed by incubation with the primary antibody for 45 min at room temperature.

Primary antibodies were diluted at an appropriate ratio in PBS + 1% bovine serum albumin/PBS. Primary antibodies were detected with EnVision conjugated with peroxidase (DakoCytomation, Hamburg, Germany) for 30 min. The substrate reaction for peroxidase was performed with the AEC substrate (DakoCytomation) according to the manufacturer’s instructions. Subsequently, the sections were washed in water, counterstained in 50% hemalum (Merck, Darmstadt, Germany) and mounted with glycerol-gelatin (Merck). The same protocol was performed for negative controls, in which an isotype-matched control antibody was used, which revealed either no staining or only weak background staining. Ki-67 antibody was bought from IMMUNOTECH SAS/Beckman Coulter, Marseille, France.

#### 2.11.2. FFPE Material

The tissue samples were fixed in 4% buffered PFA and embedded in paraffin. The 3 µm thick sections were dewaxed (3× xylol 10 min, 2× ethanol 100% 5 min, 1× ethanol 96% 5 min, 1× ethanol 90% 5 min, 1× ethanol 70% 5 min, distilled H_2_O 5 min). After high temperature antigen demasking with 10 mM citrate buffer pH 6.0 (Target Retrieval Solution, S1699, DakoCytomation) at 99 °C for 25 min and cooling for 15 min at RT, the sections were blocked with blocking solution (Zytomed, Berlin, Germany) and washed for 5 min with PBS. All incubations were carried out in a moist chamber. The following antibodies and isotype control were used for IHC:

Isotype control rabbit (monoclonal rabbit IgG, Abcam, Cambridge, UK), anti-CD95/sCD95Fc (rabbit polyclonal IgG, affinity purified, Apogenix, GmbH, Heidelberg, Germany) not reacting with murine CD95, anti-CD95L (rabbit monoclonal antibody raised against an NT-peptide of CD95L, clone 24, Apogenix) positive for human and murine CD95 ligand. The sections were incubated with antibodies diluted in antibody diluent (Zytomed) for 60 min at room temperature, washed twice in PBS, incubated with ZytoChem Plus AP Polymer anti-rabbit (Zytomed) for 30 min at room temperature and rinsed twice for 5 min in PBS. After that the sections were incubated with substrate (liquid permanent red, DakoCytomation). The reaction was stopped with distilled H_2_O. The sections were counterstained with hematoxylin (Merck).

### 2.12. Statistical Analysis

All statistical analyses were performed using Microsoft Excel and the statistical software GraphPad Prism 5 (GraphPad Software, San Diego, CA, USA) or IBM-SPSS-Statistics V.25 (IBM, München, Germany). Continuous variables were expressed as mean ± SEM. The differences among the groups were tested by Student’s *t*-test (two-tailed unpaired *t*-test), or in case of non-parametric data, the Mann–Whitney U-test. Normal distribution of the data was tested by the Shapiro–Wilk method. *p* values smaller than 0.05 were considered statistically significant and indicated on corresponding data as asterisks. Real time PCR data were analyzed using the StepOne Plus software (Thermo Fisher Scientific, Germany) and the qBase analytical software (Biogazelle, Zwijnaarde, Belgium). Analysis of the FACS data for histogram plots, dot plots and fluorescence intensity was carried out by using the free flow cytometry software WinMDI-2.8 (distributed by the Purdue University, West-Lafayette, IN, USA). For the proliferation assays, mean values and SEM were calculated from 4-fold determinations.

## 3. Results

### 3.1. Expression of CD95 and CD95L in Pancreatic Tumor Cell Lines

**Figure 1:**
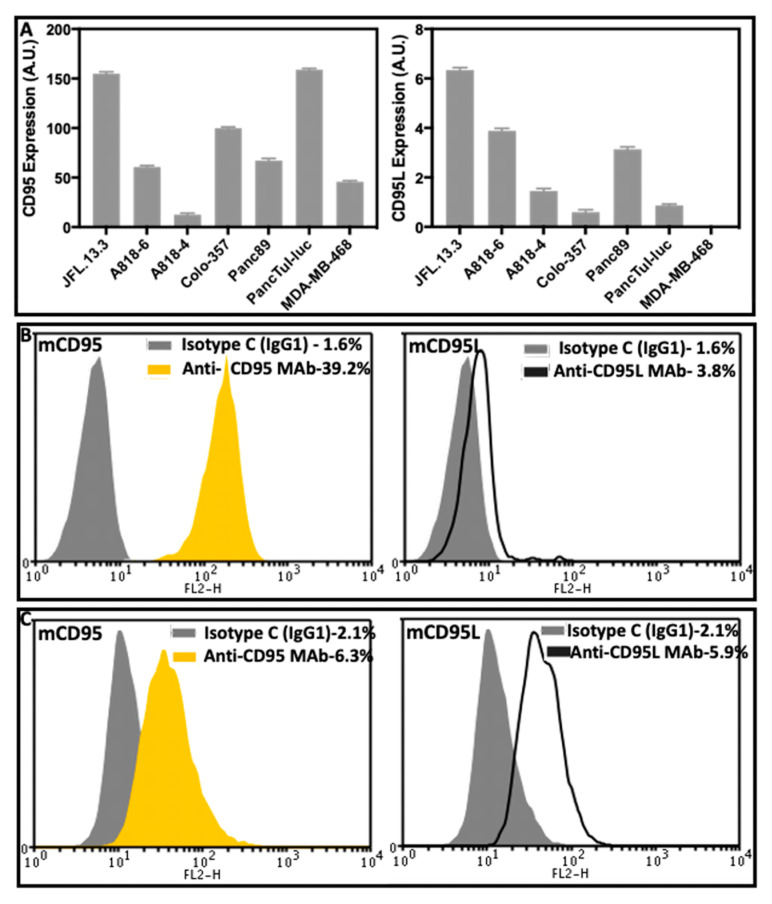
Expression of CD95 and CD95L in different PDAC cell lines. The human pancreatic cancer cell lines A818-6, A818-4, Colo-357, Panc89 and PancTuI-luc including one CD95L-OE T-lymphocyte cell line-JFL.13.3 and one breast cancer cell line-MDA-MB-468 (negative control for CD95L) were seeded at 2.8 × 10^5^/well in 6-well plates. (**A**) Cells were lysed in RNA Lysis Buffer T, total RNA was isolated, and assayed for CD95 and CD95L via PerfectProbe real-time PCR Assay. Relative expression was normalized to the control and reference gene ATP5B for CD95 and RPL13A/SDHA for CD95L and expressed as arbitrary unit (AU). Data represent means ± SD of technical replicates (*n* = 3–4). (**B**,**C**) m(membrane)CD95 and mCD95L cell surface expression was assayed in nonpermeabilized pancreatic tumor cell lines PancTuI-luc (**B**) and A818-4 (**C**) using phycoerythrin-conjugated mouse anti-human-CD95 antibody DX-2 (yellow filled peaks) and mouse anti-human-CD95L antibody NOK-1 (black line peaks), or an irrelevant antibody (IgGl isotype control) used for the determination of background staining (gray filled peaks). The raw data were plotted as histograms and representative histograms (*n* = 2) of flow cytometry analyses of PDAC cells are shown.

CD95 expression has previously been demonstrated in surgical specimens as well as in PDAC cell lines [38,39], but the expression of CD95L in PDAC cell lines has not been characterized in detail. To investigate whether the PDAC cell lines used in this experiment possess the ability to synthesize CD95 and CD95L mRNA, we performed semi-quantitative gene expression analyses in five different PDAC cell lines (A818-6, A818-4, Colo-357, Panc89 and PancTuI-luc) and a T-lymphocyte cell line, JFL.13.3 (positive control for CD95L), and a breast cancer cell line, MDA-MB-468 (negative control for CD95L), by means of a PerfectProbe^®^ PCR assay. Both CD95 and CD95L mRNA were detected in all five PDAC cell lines as well as in JFL.13.3. MDA-MB-468 cells showed moderate CD95-, but no CD95L expression. We observed a differential expression pattern for both CD95 and CD95L in the PDAC cell lines. However, comparing the expression levels of our two main model cell lines A818-4 and PancTuI-luc, we noted that the expression of CD95 was high in PancTuI-luc compared to A818-4 while the pattern got reverse for the expression of CD95L (Figure 1A).

Even though surface CD95 was highly expressed in most of the PDAC cell lines, the expression of surface CD95L was either very low or undetectable, possibly due to cleavage by metalloproteinases. Since several previous reports confirmed that the blockade of MMP7 and ADAM17 increased surface expression of CD95L, we also used two different types of broad range MMP inhibitors, Ilomastat and Batimastat, to treat our experimental cell models (PancTuI-luc and A818-4) to increase the expression level of surface CD95L. Here, we observed a moderate stimulation of surface expression of CD95L by 1.2-FC and CD95 by 1.0-FC with Ilomastat while CD95L expression slightly increased by 1.9-FC and CD95 decreased by 1.3-FC with Batimastat treatment in PancTuI-luc (Supplementary Figure S1A). Neither of the treatments showed any considerable effect on the surface expression of CD95 (slight decrease by 0.92-FC) in the A818-4 cell line while a modest reduction was noted on the expression of CD95L by 0.7-FC and 0.8-FC with Ilomastat and Batimastat, respectively (Supplementary Figure S1B).

Since these findings show only marginal effects on the expression of both CD95 and CD95L in our cell model system, we preferred not to include the additional treatment with such MMP inhibitors in our mainstream experiments, assuming there could be some additional shedding mechanisms of CD95 and CD95L. Furthermore, we were able to analyze the surface expression of both CD95 and CD95L in our model cell lines (PancTuI-luc and A818-4) without using any metalloproteinase inhibitors. Here, we have detected a strong surface expression of CD95, while the surface expression level of CD95L was low mainly in PancTuI-luc cells, but sufficient to detect and induce pro-inflammatory and pro-survival signaling (Figure 1B,C).

### 3.2. Impact of CD95L Inhibition on the Gemcitabine Induced Inflammatory Responses

In previous studies, we have found, along with others, that chemotherapeutic agents enhanced the surface expression of CD95. Here, we analyzed the membrane-bound protein as well as mRNA expression of both CD95 and CD95L in experimental tumors in vivo as well as in the pancreatic tumor cells in vitro after treatment with gemcitabine in connection with non-apoptotic functions. We also analyzed the impact of gemcitabine treatment on the expression of several tumor-promoting genes-TPGs (IL8, IL6, CD44 and CD133) as well as CD95L in experimental tumors derived from the PancTuI-luc cell line with two different settings of treatment (palliative and adjuvant). We detected that gemcitabine (5 µg/g-bwt) significantly induced the expression of all TPGs in the experimental tumors, with the exception of CD44 expression in a palliative setting (Supplementary Figure S2A,B). Importantly, CD95L mRNA expression was also found to be increased under both conditions, suggesting a combination of the inhibitor sCD95Fc together with gemcitabine in the future (Supplementary Figure S2C,D).

**Figure 2:**
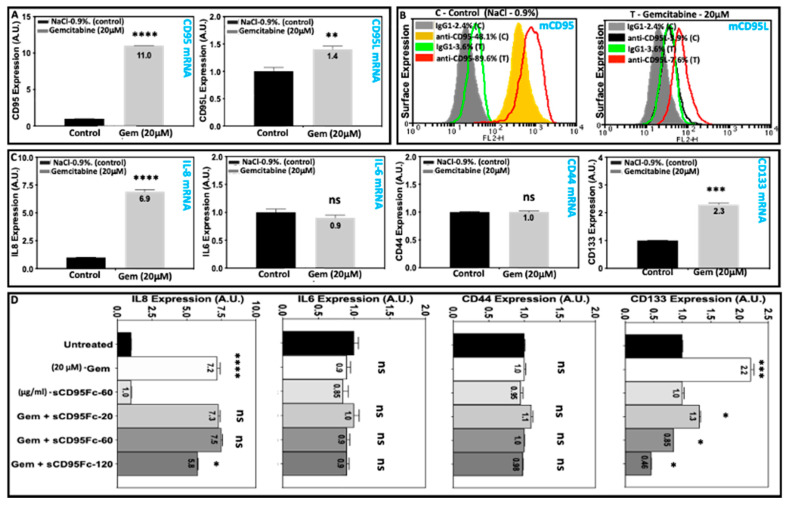
Inhibitory effects of sCD95Fc on the gemcitabine-induced expression of PICs (IL8 and IL6), and PCSC markers (CD44 and CD133) in pancreatic tumor cells. Human PDAC cell line PancTuI-luc was seeded with 2.8 × 10^5^ cells/well in a 6-well plate. Cells were either treated with 0.9 % saline (**A**–**C**) or untreated (**D**) as controls or treated with gemcitabine (20 μM) alone (**A**,**C**) or in combination with three different concentrations of sCD95Fc (20, 60, 120 μg/mL) (**D**). Cells were lysed in RNA Lysis buffer T, total RNA was isolated, and assayed for CD95 and CD95L (A, upper row) or IL8, IL6, CD44 and CD133 (**C**,**D**) via PerfectProbe real-time RT-PCR assays. Relative expression was normalized to the control and reference gene UBC and expressed as arbitrary unit (AU). Controls were set as 1 in each experiment. Data represent means ± SD of biological replicates (*n* = 2) with three technical replicates each. In case of statistical significance, asterisks indicate the corresponding significance levels (Student’s *t*-test; *, *p* < 0.05; **, *p* < 0.01; ***, *p* < 0.001; ****, *p* < 0.0001; ns, not significant). (**A**,**C**,**D**). B: mCD95 and mCD95L cell surface expression was assayed in non-permeabilized PancTuI-luc using phycoerythrin-conjugated mouse anti-human-CD95 antibody DX-2 (filled yellow peak-untreated and Red line peaks-treated with Gemcitabine (20 μM) and mouse anti-human-CD95L antibody NOK-1 (black line peaks-untreated and red line peaks -treated with gemcitabine (20 μM) or an irrelevant antibody (IgGl isotype control) used for the determination of background staining (grey filled peaks-untreated and green line peaks-treated with gemcitabine (20 μM). Raw data were evaluated as histogram plots as described in Section 2.

To test whether gemcitabine would have similar impact on the induction of these TPGs (IL8, IL6, CD44 and CD133) in our in vitro system under the influence of CD95 signaling, we first analyzed the effect of gemcitabine treatment on the mRNA as well as surface protein expression of CD95 and CD95L. It could be noted that the mRNA expression of CD95 was increased by 11-fold, while CD95L was enhanced by only 1.4-fold upon gemcitabine treatment (20 µM) compared to the untreated (saline control-0.9%NaCl) pancreatic tumor cell line PancTuI-luc (Figure 2A). The respective protein expression at the cell surface of CD95 was enhanced by 1.9-fold change (FC), while CD95L was upregulated by 2.7-FC after gemcitabine treatment (20 µM) compared to the surface expression of CD95 and CD95L in the untreated pancreatic tumor cells (Figure 2B).

Furthermore, we analyzed the expression of TPGs (IL8, IL6, CD44 and CD133) after the treatment with gemcitabine (20 μM) for 24 h in the same PDAC cell line PancTuI-luc. In these cells, IL8 and CD133 were found to be induced by 6.9 and 2.3-fold, respectively, after 24 h of gemcitabine treatment. However, no effects on the expression of both IL6 and CD44 were noted (Figure 2C). Finally, we analyzed the expression of the same panel of TPGs (IL8, IL6, CD44 and CD133) in PancTuI-luc cell line upon the treatment with gemcitabine in combination with three different concentrations of CD95L inhibitor (sCD95Fc-20, 60, 120 μg/mL) for 24 h. Cells left untreated served as controls. Again, it was noted that inhibition of CD95L by sCD95Fc exerts inhibitory effects on the gemcitabine-induced expression of IL8 and CD133, but not of IL6 and CD44 in a dose and target-dependent manner after 24 h of treatment (Figure 2D). One possible bias in this setting is the gemcitabine impact on the CD95 system. In Supplementary Figure S3 we show a profound enhancement of CD95 expression upon 24 h treatment with gemcitabine and a significant reduction in the combined treatment with sCD95Fc, indicating a contribution of autocrine stimulation. CD95L expression was also affected, yet to a lesser extent.

### 3.3. Inhibition of CD95L Exerts Synergistic Effects on the Gemcitabine Induced Cell Death of Pancreatic Tumor Cells

Next, the impact of CD95L blockade on the gemcitabine-induced cell death was investigated using the two PDAC cell lines PancTuI-luc and A818-4.

**Figure 3:**
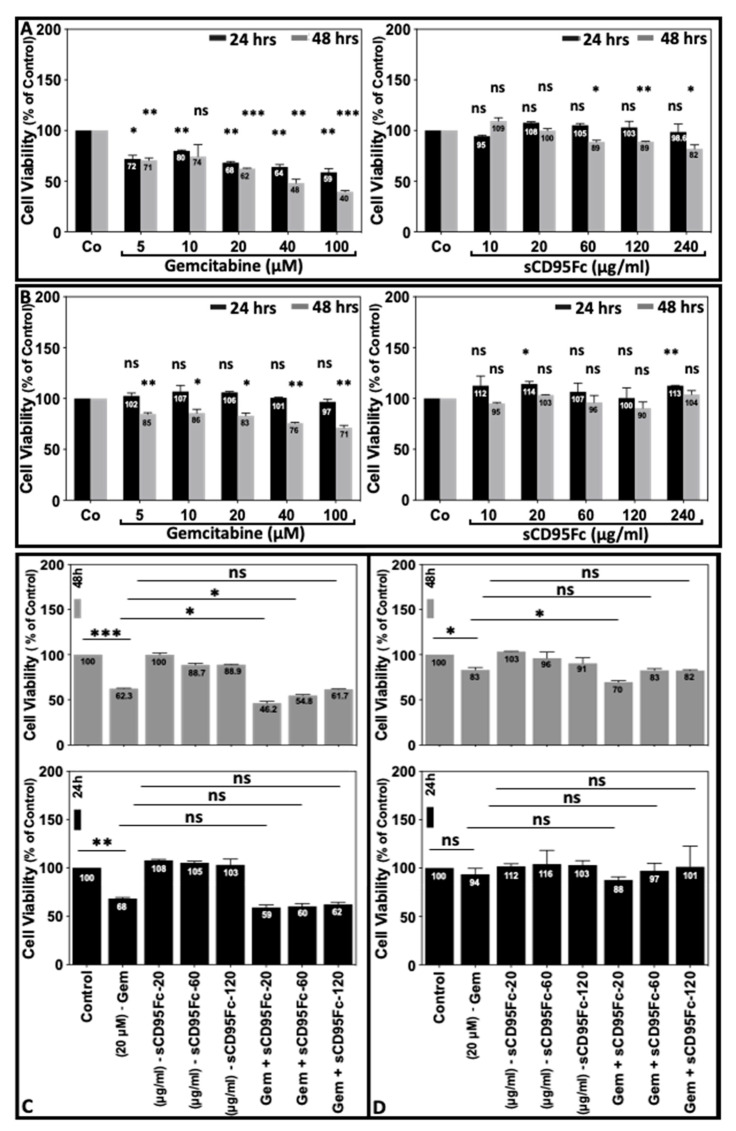
Effect of CD95L inhibition on the gemcitabine-induced cell death of pancreatic tumor cells. Human PDAC cells PancTuI-luc (**A**,**C**) and A818-4 (**B**,**D**) were seeded in standard cell culture medium in 96-well plates at densities of 1.0 × 10^4^ for a 24 h treatment or 0.7 × 10^4^, respectively, for a 48 h treatment. After initial growth for 24 h, the medium was replaced with fresh medium (100 μL/well) with escalating concentrations (0 [Control, Co], 5, 10, 20, 40, 100 μM) of gemcitabine or (0 [Control, Co], 10, 20, 60, 120, 240 μg/mL) of sCD95Fc (**A**,**B**) or gemcitabine (Gem-20 μM) alone or with three different concentrations of sCD95Fc (20, 60, 120 μg/mL) (**C**,**D**) for either 24 h (black bars) or 48 h (grey bars). The effect of chemotherapeutic agent and death ligand inhibitors on the cell proliferation was tested by WST-1 assays and is depicted as cell viability as percent of controls (100% untreated cells with WST-1 reagent). Statistical analysis (Student’s *t*-test) was performed between controls vs gemcitabine (Gem) and Gem vs Gem+sCD95Fc. Each bar represents the mean of two independent experiments (*n* = 2) with four parallel determinations each, error bars indicate the corresponding SDs. Asterisks indicate statistical significance levels (* *p* < 0.05; ** *p* < 0.01; *** *p* < 0.001; ns, not significant).

The cells were treated with escalating doses of gemcitabine (5, 10, 20, 40, 100 μM) and sCD95Fc (10, 20, 60, 120, 240 μg/mL) for 24 h and 48 h. WST-1 cell proliferation assays were performed to evaluate the sensitivity and resistance towards apoptosis of these agents. Dose-response results of the percentage of cell viability are shown in Figure 3A,B.

It was found that the PancTuI-luc cell line was clearly more sensitive than A818-4 cells towards gemcitabine-induced cell death, with the latter showing either no or very low inhibition (at the highest gemcitabine concentration) after 24 h of treatment. The increasing concentrations of gemcitabine decreased the cell viability of PancTuI-luc cells in a dose-dependent manner after both, 24 h and 48 h of treatment leading to 59% and 40% viability, respectively. After the 48 h treatment, also A818-4 cells exhibited a moderate decrease in cell viability to 71% with the highest gemcitabine concentration (Figure 3A,B, left). Regarding a potential suicidal effect of autocrine CD95L, we did not observe any significant changes in the growth rate of both the cell lines after treatment with different concentrations of CD95L-blocking sCD95Fc (Figure 3A,B, right).

Further, we analyzed the impact of CD95L inhibition on the sensitization against gemcitabine-induced cell death in both the cell lines. It could be noted that gemcitabine alone decreased the cell viability by 32% in PancTuI-luc (*p* < 0.01) and not significantly in A818-4 cells at 24 h and by 37.7% (*p* < 0.001) and 17 % (*p* < 0.05) at 48 h of treatment in PancTuI-luc and A818-4, respectively. Gemcitabine treatment combined with inhibition of CD95L by sCD95Fc (20 μg/mL) further decreased the cell viability by 16.1% and 13% in PancTuI-luc and A818-4 cell lines, respectively, after 48 h of treatment, compared with gemcitabine alone (*p* < 0.05; Figure 3C,D, upper panel), whereas higher doses of the inhibitor did not improve this combined effect.

### 3.4. Effects of Death Ligands on the Cell Proliferation of PDAC Cells

**Figure 4:**
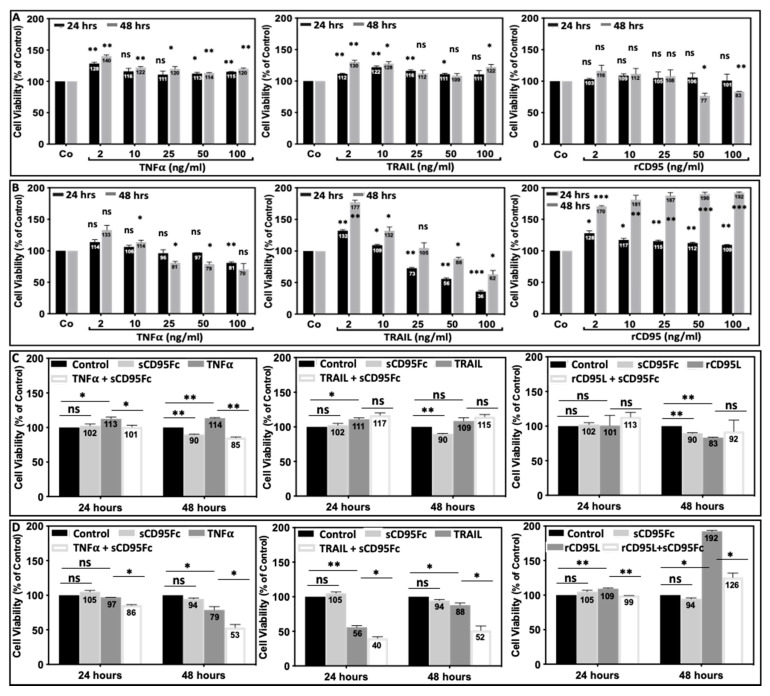
Death ligands (TNF-α, TRAIL, CD95L) affect pancreatic tumor cell growth. Human pancreatic adenocarcinoma cells PancTuI-luc (**A**,**C**) and A818-4 (**B**,**D**) were seeded in 96-well plates at a density of 1.0 × 10^4^ or 0.7 × 10^4^ for treatments of 24 h and 48 h, respectively and were grown in standard cell culture medium. After a 24 h incubation the medium was replaced with fresh medium (100 μL/well) with escalating amount (2, 10, 25, 50, 100 ng/mL) of TNF-α, TRAIL and recombinant (r)CD95L (**A**,**B**) or TNF-α ± sCD95Fc (60 μg/mL) or TRAIL ± sCD95Fc (60 μg/mL) or rCD95L ± sCD95Fc (60 μg/mL) (**C**,**D**) for 24 h and 48 h. The effect of death ligands on cell proliferation was analyzed by WST-1 proliferation assays and is depicted as cell viability. The absorbance was measured at 460 nm in microplate reader and was calculated as percent of controls (untreated cells). Statistical analysis (Student’s *t*-test) was performed comparing controls vs. sCD95Fc or TNF-α/TRAIL/rCD95L and TNF-α/TRAIL/rCD95L vs TNF-α/TRAIL/rCD95L +sCD95Fc. Each bar represents the mean of two independent experiments (*n* = 2) with four parallel determinations each, error bars indicate the corresponding SDs. Asterisks indicate statistical significance levels (* *p* < 0.05; ** *p* < 0.01; *** *p* < 0.001; ns, not significant).

Two PDAC cell lines PancTuI-luc and A818-4 were treated with escalating concentrations of TNFα or TRAIL or rCD95L (2, 10, 25, 50, 100 ng/mL) for 24 h and 48 h. WST-1 cell proliferation assays were performed to measure cell proliferation and to evaluate the sensitivity or resistance of these PDAC cell lines against death ligands-induced cell growth stimulation. The PancTuI-luc cell line clearly exhibited a resistant phenotype (rather moderate to strong growth stimulation than apoptosis) by treatment with all three death ligands, TNF-α, TRAIL and rCD95L. Only the two highest concentrations of rCD95L induced a moderate growth reduction and only after 48 h. In contrast, the more sensitive cell line A818-4 exhibited a moderate to strong growth reduction towards TNF-α and more pronounced against TRAIL but showed moderate to strong growth stimulation against rCD95L treatment both after 24 h and 48 h (Figure 4A,B left, middle, right). Remarkably, after 48 h, a strong increase of cell growth (up to 192%) of A818-4 cells with all concentrations of rCD95L treatment was found that indicates a particular aggressiveness of these cells in response to rCD95L.

Table 1 briefly summarizes the findings of this analysis of sensitivity or resistance of the cell lines PancTuI-luc and A818-4 towards death ligands and gemcitabine.

Further, the two PDAC cell lines, PancTuI-luc and A818-4, were left untreated or treated with three different death ligands (TNF-α, TRAIL, CD95L) in the presence or absence of sCD95Fc (60 μg/mL) for 24 h and 48 h. It was found that sCD95Fc neutralized the growth stimulatory effects of TNF-α in PancTuI-luc, while it showed additive effects on the growth reduction of TNF-α in A818-4 both after 24 h and even stronger after 48 h of treatment (Figure 4C,D; left). In contrast to the effects of TNF-α, it emerged that the A818-4 cell line exerted even more prominent effects on the TRAIL-induced growth reduction when combined with sCD95Fc (Figure 4D middle) while non-significant effects were noted on cell viability of PancTuI-luc after treatment with TRAIL + sCD95Fc compared to TRAIL alone at both, 24 h and 48 h (Figure 4C, middle). In addition, the A818-4 cell line showed strong proliferative effects upon rCD95L treatment (Figure 4D right), while the cell viability is even decreased with rCD95L stimulation in PancTuI-luc cell line at 48 h (Figure 4C right). Further, we detected that the treatment with sCD95Fc effectively neutralized the proliferative effects of rCD95L in A818-4 cell line while no effect was observed for PancTuI-luc cell line after 48h (Figure 4C,D right). Altogether, these findings indicate that the treatment with sCD95Fc increases the effect of TNF-α and TRAIL while it neutralizes the effect of rCD95L in the A818-4 cell line. In case of PancTuI-luc, sCD95Fc only nullified the growth stimulatory effects of TNF-α while no effect was observed with TRAIL or rCD95L. The different response of these death ligands in these two PDAC cell lines is probably due to the varying level of sensitivity/resistivity of these death ligands towards these cell lines as shown in Table 1.

### 3.5. Effect of CD95L Inhibition on the Expression of CD95 and CD95L Induced upon TNF-α, TRAIL and rCD95L Stimulation

**Figure 5:**
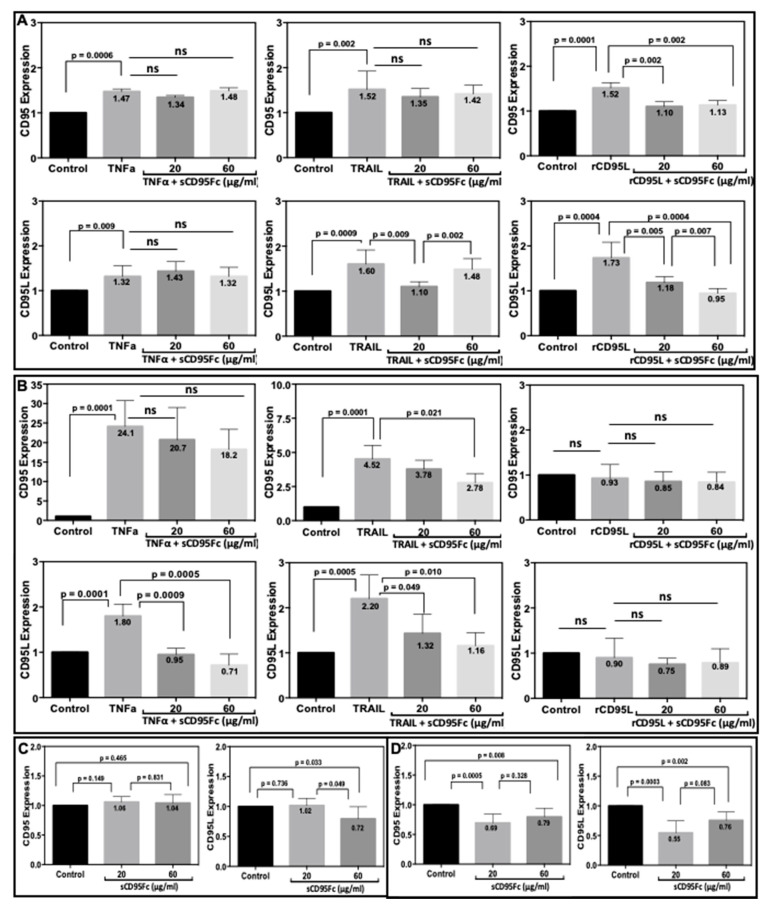
Inhibitory effects of sCD95Fc on CD95 and CD95L gene expression in pancreatic cancer cell lines upon TNF-α, TRAIL and rCD95L treatment. Human pancreatic cancer cells PancTuI-luc (**A**,**C**) and A818-4 (**B**,**D**) were seeded with 2.8 × 10^5^ cells/well in 6-well plates. Cells were either left untreated (control; black bar) or stimulated with 50 ng/mL TNF-α or 50 ng/mL TRAIL or 100 ng/mL rCD95L (grey bar), TNF-α ± sCD95Fc (20 μg/mL) or TRAIL ± sCD95Fc (20 μg/mL) or rCD95L ± sCD95Fc (20 μg/mL) (dark grey) and TNF-α ± sCD95Fc (60 μg/mL) or TRAIL ± sCD95Fc (60 μg/mL) or rCD95L ± sCD95Fc (60 μg/mL) (light grey) or treated alone with sCD95Fc (20 μg/mL; light grey) or (60 μg/mL; dark grey) for 36 h. Cells were lysed in RNA Lysis Buffer T; total RNA was isolated and assayed for CD95 (upper row; **A**,**B** or left panel; **C**,**D**) and CD95L (lower row; **A**,**B** or right panel; **C**,**D**) by PerfectProbe realtime-RT-PCR Assay. Relative expression was normalized to the control and reference genes UBC or RPL13A and expressed as arbitrary unit (AU). Controls were set as 1 in each experiment and normal distribution of the data was tested by the Shapiro–Wilk method. *p*-values indicate statistical significance levels, while non-significant data represented by ns.). Data represent means ± SEM of biological replicates (*n* = 3).

CD95 and CD95L as transmembrane proteins are key molecules of this crosstalk study. Therefore, we investigated the impact of TNF-α, or TRAIL, or rCD95L signaling on the expression of CD95 and CD95L expression in the presence or absence of the CD95L inhibitor sCD95Fc (Figure 5). We found that rCD95L (100 ng/mL) stimulation elevates the expression of both CD95 and CD95L in PancTuI-luc cells (Figure 5A right). Pre-treatment with sCD95Fc significantly neutralized this effect at 36h of treatment. However, we did not observe any such effects of the same treatment in the A818-4 cell line model (Figure 5B right). This experiment was used as a positive control for the crosstalk of TNF-α, or TRAIL with CD95L. Stimulation with TNF-α or TRAIL (50 ng/mL) significantly up-regulated the expression of both, CD95 and CD95L in PancTuI-luc (Figure 5A left, middle) and was even more pronounced in A818-4 cells (Figure 5B left, middle). The pre-incubation with sCD95Fc exerted varying effects showing either no significant neutralizing activity on CD95 expression after TNF-α or TRAIL treatment in PancTuI-luc (Figure 5A upper left, middle) or a partial neutralization of TRAIL-effects on CD95 expression in A818-4 cells (Figure 5B upper middle). Moreover, no significant neutralization by sCD95Fc of the high CD95 induction by TNF-α could be observed in this cell line (Figure 5B upper left). Regarding CD95L expression, significant neutralization of the effects of both, TNF-α and TRAIL after preincubation with sCD95Fc were found in A818-4 cells (Figure 5B lower left, middle) as well as in PancTuI-luc cells in the case of TRAIL stimulation (Figure 5A lower middle). However, no neutralizing effect of sCD95Fc was observed in this cell line with regard to the CD95L stimulation by TNF-α (Figure 5A lower left). In addition, we analyzed the effects of CD95L inhibition by two concentrations (20 and 60 µg/mL) of sCD95Fc on the constitutive expression of CD95 and CD95L. Blocking of CD95L led to a significant reduction of the constitutive expression of both, CD95 and CD95L by 1.5-fold (*p* = 0.0005) and 1.8-fold (*p* = 0.0003) respectively, in the A818-4 cell line after 36 h of sCD95Fc treatment (20 µg/mL, Figure 5D). By contrast, in PancTuI-luc cells, only inhibition of CD95L with the high concentration of sCD95Fc showed an inhibitory effect on the constitutive expression of CD95L by 1.4-fold (*p* = 0.033, Figure 5C). Here, we can conclude that both TNF-α and TRAIL can induce both CD95 and CD95L in both PDAC cell lines, while the modulating/neutralizing effects of sCD95Fc on the stimulatory activity of TNF-α and TRAIL are mainly detected in the TNF-α and TRAIL-sensitive cell line (A818-4), possibly due to strong proinflammatory signaling upon the induction of both CD95 and CD95L. However, the effects of rCD95L alone or in combination with sCD95Fc were detected only in CD95L-sensitive cell line i.e., PancTuI-luc cells as rCD95L shows no effect in A818-4.

**Figure 6:**
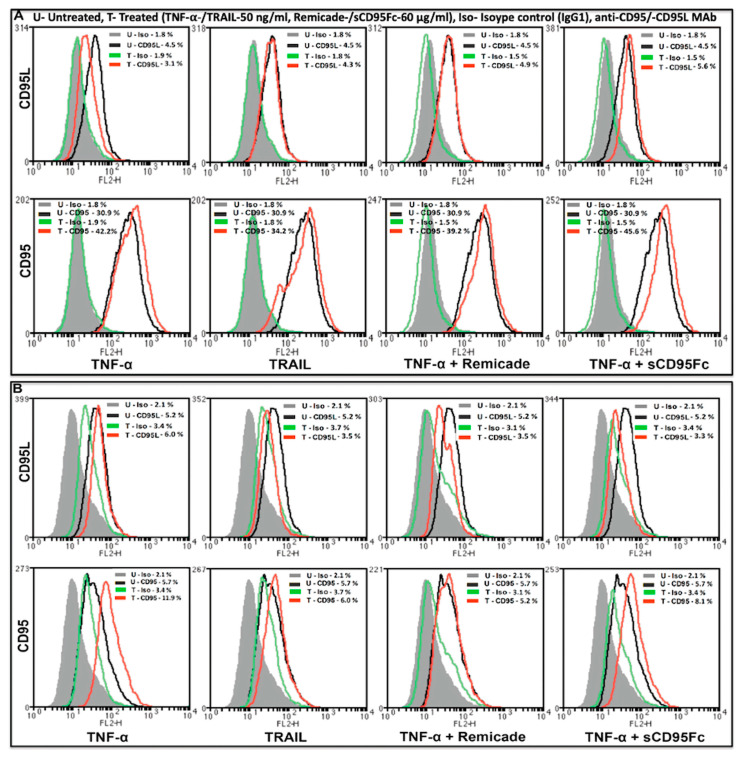
Analysis of PDAC cell-membrane-bound CD95L (**A**,**B** upper row) and CD95 (**A**,**B** lower row) protein expression by flow cytometry after treatment with TNF-α or TRAIL. CD95L and CD95 cell surface expression was assayed in non-permeabilized PancTuI-luc cells (**A**) and A818-4 cells (**B**) using phycoerythrin-conjugated mouse anti-human-CD95 antibody DX-2 or mouse anti-human-CD95L antibody NOK-1 (black line peaks -untreated and red line peaks -treated with TNF-α or TRAIL ± Remicade or sCD95Fc) or an irrelevant antibody (IgGl isotype control) used for the determination of background staining (grey filled peaks-untreated and green line peaks-treated with TNF-α or TRAIL ± Remicade or sCD95Fc). Raw data were analyzed and evaluated as histogram plots (*n* = 2) as described in Section 2.

Further, the effects of TNFα and TRAIL on the cell surface expression of CD95 (lower row)- and CD95L (upper row) proteins were analyzed by flow cytometry in the PDAC cell lines, PancTuI-luc (Figure 6A) and A818-4 (Figure 6B). It was found that stimulation with TNF-α (50 ng/mL) slightly decreased the expression of mCD95L by 0.4-FC in PancTuI-luc and 0.7-FC in A818-4, while it up-regulated the expression of mCD95 by 1.4-FC in PancTuI-luc and 2.4-FC in A818-4 after 36 h. Additionally, the treatment with TRAIL (50 ng/mL) also slightly to moderately decreased the expression of mCD95L by 0.9-FC in the PancTuI-luc cell line and 0.06-FC in the A818-4 cell line after 36 h of treatment. In contrast, TRAIL slightly enhanced the surface expression of CD95 by 1.1-FC in the PancTuI-luc cell line it while slightly reduced it by 0.7-FC in the A818-4 cell line after 36 h of treatment. Additionally, we analyzed the neutralizing effects of Remicade (anti-TNF-α antibody “infliximab”) on the surface expression of CD95 and CD95L. We observed that pre-treatment with Remicade or sCD95Fc along with TNF-α decreased/nullified the impact of exogenous TNF-α-induced modulation of mCD95/mCD95L, both in PancTuI-luc and A818-4 cell lines. Interestingly, pre-treatment with Remicade/sCD95Fc showed a neutralizing effect by increasing 1.3-FC or 1.5-FC expression of mCD95L while a marginal effect on the TNF-α-induced mCD95 expression by again 1.3-FC or 1.5-FC in the PancTuI-luc cell line. Contrastingly, an inverse result was reported in case of the A818-4 cell line where the pre-treatment with Remicade/sCD95Fc showed a synergizing effect on the mCD95L expression by a decrease of 0.13-FC or 0.03-FC, while a nullifying effect was shown on the mCD95 expression by a decrease of 0.6-FC or increase of 1.3-FC upon TNF-α stimulation for 36 h (Figure 6). Despite the seemingly impressive impact of these pre-treatments, the overall impact is low, likely due to the protease-mediated cleavage of mCD95 and mCD95L.

### 3.6. Effect of CD95L Inhibition on the Expression of IL8 and IL6 Induced upon TNF-α, TRAIL and CD95L Stimulation

**Figure 7:**
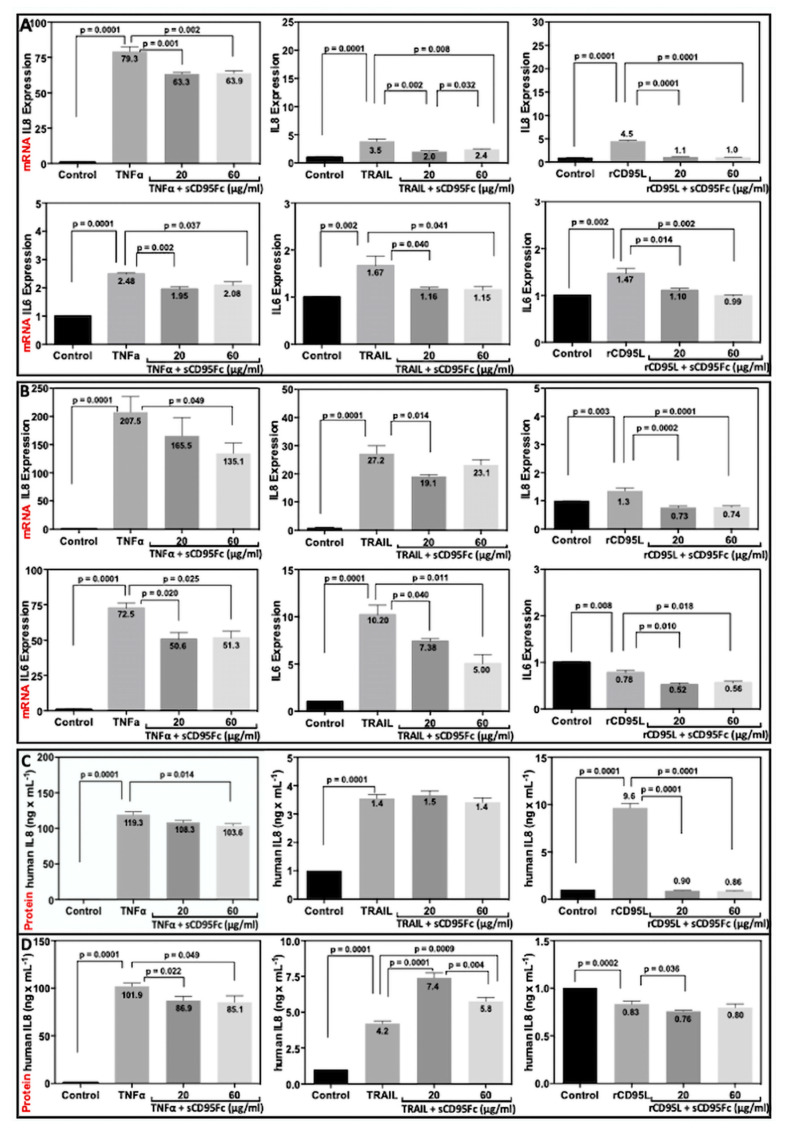

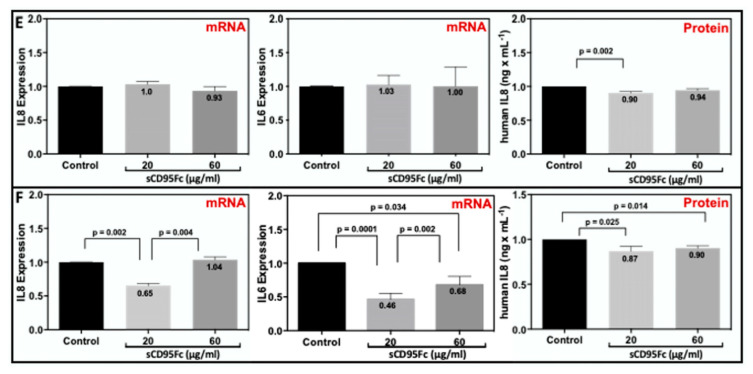
Inhibitory effects of sCD95Fc on IL8 and IL6 mRNA and protein expression in pancreatic cancer cell lines upon TNFα, TRAIL and rCD95L treatment. Human pancreatic cancer cells PancTuI-luc (**A**) and A818-4 (**B**) were seeded with 2.8 × 10^5^ cells/well in 6-well plates. Cells were either left untreated as control (black bar) or stimulated with 50 ng/mL TNF-α or 50 ng/mL TRAIL or 100 ng/mL rCD95L (grey bar), TNF-α ± sCD95Fc (20 μg/mL) or TRAIL ± sCD95Fc (20 μg/mL) or rCD95L ± sCD95Fc (20 μg/mL) (dark grey) and TNF-α ± sCD95Fc (60 μg/mL) or TRAIL ± sCD95Fc (60 μg/mL) or rCD95L ± sCD95Fc (60 μg/mL) (light grey) for 36 h. Cells were lysed in RNA Lysis Buffer T; total RNA was isolated, and assayed for IL8 (upper) and IL6 (lower) by PerfectProbe RT-PCR assays (**A**,**B**). Relative expression was normalized to the control and reference genes UBC or RPL13A and expressed as arbitrary unit (AU). (**C**,**D**) After the indicated time point (36 h), cell supernatants of PancTuI-luc (**C**) and A818-4 cells (**D**) was collected and centrifuged at 14,000 rpm at 4 °C for 15 min in an Eppendorf centrifuge to remove debris and dead cells and cleared supernatants were further analyzed by using a human IL8 ELISA. PancTuI-luc (**E**) and A818-4 (**F**) cells were treated with sCD95Fc (20 μg/mL) (light grey) or (60 μg/mL) (dark grey) for 36 h and then IL8 mRNA (left and middle panel) and IL8 protein (right panel) were analyzed. Controls were set as 1 in each experiment and the normal distribution of the data was tested by Shapiro–Wilk method. In case of significance, *p*-values were indicated. Data represent means ± SEM of biological replicates (*n* = 3).

To further characterize the death ligand cross-talk system in the PDAC model cell lines PancTuI-luc and A818-4, we analyzed the dose-dependent effects of TNF-α, TRAIL and CD95L on the expression of PICs (IL8 and IL6) by real-time PCR assays. It could be observed that TNF-α has strong dose-dependent effects (data not shown) on the induction of both IL8 and IL6 mRNA expression as well as IL8 protein secretion in comparison to TRAIL and rCD95L in both the cell lines (Figure 7A–D). Furthermore, the stimulatory effects of these death ligands were more pronounced in A818-4 than in PancTuI-luc.

Based on the previous characterization, we chose the optimum concentration of (50 ng/mL) for TNF-α or TRAIL and (100 ng/mL) for rCD95L stimulation and analyzed the effects of CD95L inhibition on the TNF-α or TRAIL-induced IL8 and IL6 expression after 36 h of stimulation in the same panel of cell lines. In PancTuI-luc cell line, we observed that rCD95L induced the expression of both IL8 (by 4.5-fold) and IL6 (by1.5 fold) upon 36 h of stimulation and this effect was efficiently neutralized after a pre-treatment with sCD95Fc. Further, it was also noted that the expression of both IL8 and IL6 was induced by both TNF-α (79.3-fold and 2.5-fold) and TRAIL (3.5-fold and 1.7-fold), respectively and the blockade of CD95L using sCD95Fc exerted a considerable inhibitory effect on the expression of both IL8 and IL6 induced upon TNF-α and TRAIL stimulation after 36 h (Figure 7A). In case of the A818-4 cell line, we detected an even stronger upregulation of IL8 and IL6 compared to the PancTuI-luc cell line, except for the expression of IL6 upon rCD95L stimulation, which showed a slight decrease. However, after the strong induction of IL8 and IL6 in A818-4 upon TNF-α (by 207.5-fold and 72.5-fold, respectively) or TRAIL (by 27.2-fold and 10.2-fold, respectively) stimulation, the inhibitory effects of sCD95Fc was less significant as in the PancTuI-luc cell line after 36 h of treatment (Figure 7B).

Since the PDAC cells also showed autocrine synthesis of TNF-α, TRAIL and CD95L, the impact of CD95L inhibition on endogenous IL8 and IL6 was analyzed without any exogenous stimulation in PDAC cells. We noted that the treatment of sCD95Fc exerts no effect on the expression of both, IL8 and IL6 after 36 h of treatment in PancTuI-luc cells, while significant inhibitory effects on the expression of both IL8 and IL6 mRNA were observed with a stronger effect of the lower concentration of sCD95Fc after 36 h of treatment in the A818-4 cell line. However, these changes in A818-4 cells on the mRNA level were not paralleled on the protein level, where no significant modulation by sCD95Fc emerged. (Figure 7E,F, left and middle).

Further, we analyzed the secretion of IL8 protein in the cell culture supernatants by performing human IL8 ELISA under different treatment conditions in the same panel of cell lines after 36 h. rCD95L stimulated the secretion of IL8 by 9.6-fold (*p* = 0.0001) while pre-treatment of the cells with sCD95Fc effectively neutralized the effects of the rCD95L stimulation in the PancTuI-luc cell line after 36 h (Figure 7C). However, we detected a different pattern of results in case of the A818-4 cell line as rCD95L treatment exhibits no stimulatory effect, or even decreases the secretion of IL8 (Figure 7D). In addition, TNF-α or TRAIL (50 ng/mL) strongly induced the secretion of IL8 and the pre-treatment with sCD95Fc substantially reduced the secretion of IL8 induced upon TNF-α stimulation, while it exerted either no effects or slightly co-stimulatory effects on the secretion of IL8 upon TRAIL stimulation for 36 h in both the cell lines. Intriguingly, the inhibition of CD95L without any exogenous stimulation reduced the secretion of IL8 after 36 h of treatment, both in PancTuI-luc and A818-4 cells (Figure 7C–F, left).

### 3.7. In Vivo Effects of CD95L Inhibition in Pancreatic Xenotransplant Models

Having shown the strong inhibitory impact of sCD95Fc on the expression of pro-inflammatory cytokines and stem cell markers under in vitro conditions, we aimed for testing the putative therapeutic effect under appropriate in vivo conditions. Therefore, we made use of the clinically-adapted xenotransplantation model [34,35], which had been previously established by us in a robust manner, mimicking the conservative/palliative as well as the adjuvant setting [36,37,40]. The latter procedure included a partial pancreatectomy of an orthotopically implanted pancreatic tumor after injection of PancTuI-luc cells (suspended in Matrigel) into the pancreatic tail of SCID-beige mice. The details of the procedure and the time course of the therapeutic dosing are described in Section 2.

As a first step towards clinical translation of using sCD95Fc, we investigated its effect on orthotopic pancreatic tumor growth after implanting PancTuI-luc cells 28 days prior to treatment. Four different concentrations of sCD95Fc were tested according to the details as provided in the Section 2 and the typical U-shaped dose response (Supplementary Figure S4) revealed 25 μg/g-bwt as an optimal dose under these conditions.

**Figure 8:**
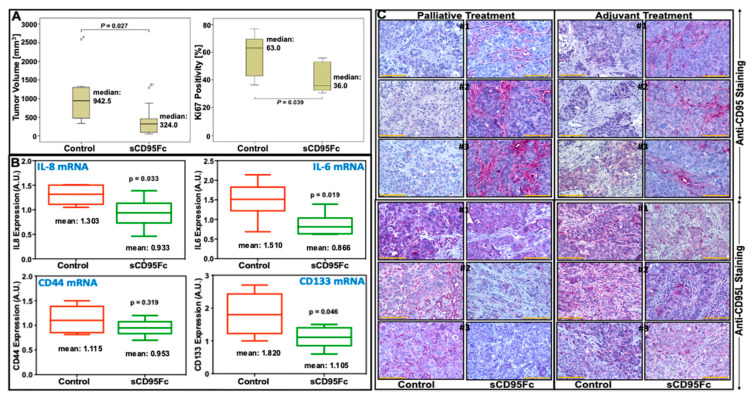
Impact of CD95L inhibition on tumor progression (tumor volume, Ki67 positivity, proinflammatory cytokines and cancer stem markers) and the CD95/CD95L immunostaining in tumors derived from PancTuI-luc cells under both palliative and adjuvant regimens. Female SCID beige mice (*n* = 9) bearing PancTuI-luc tumor xenografts were treated i.p. with either sodium chloride 0.9% (control group) or with 25 μg/g bwt of sCD95Fc (treated group). The tumor volume (**A**, left panel) of the recurrent pancreatic tumor was determined at autopsy 4 weeks after subtotal pancreatectomy in adjuvant treatment setting. The primary tumors were resected by subtotal pancreatectomy 10 d after inoculation of pancreatic tumor cells (PancTuI-luc-1 × 10^6^). Tissue cryo-sections of recurrent tumor specimens after adjuvant therapy regimen (**A**, right panel) were immunostained for Ki67 expression and the Ki67 positivity index was calculated. Ki67 positivity of individual xenograft tumors in each group. Gene expression analysis of proinflammatory cytokines (IL8 and IL6) and pancreatic stem cell markers (CD44 and CD133) was performed as indicated in Section 2 to measure the impact of CD95L inhibition in the treated tumors. The recurrent tumor tissues (*n* = 7–8) from each group were homogenized and lysed in lysis buffer; total RNA was isolated, and assayed for IL8, IL6, CD44 and CD133 via a PerfectProbe realtime RT-PCR Assay. The resulting c_T_-values of the corresponding genes were normalized against reference gene UBC and expressed as arbitrary units (AU) (**B**). Three representative examples from each treatment (25 μg/g bwt of sCD95Fc) group of the palliative and adjuvant setting are demonstrated compared to respective control animals those received 0.9% sodium chloride. Staining of the tissue sections with a human antigen-specific antibody (anti-CD95 antibody) was performed as described in Section 2. Similarly, the staining is shown for the anti-CD95L antibody, which detects both the human and the murine antigen (**C**). The orange-colored scale bars represent 100 µm. The normal distribution of the data in (8B) was tested by the Shapiro–Wilk method and in case of significance, Student’s *t*-test was performed and *p*-values and the means were indicated at the corresponding boxplots. For the tumor volumes (8A) Mann–Whitney U-tests were performed for comparison of the medians of each treated group vs. the control group.

Since the impact of inflammatory mechanisms is likely to be higher after surgical intervention, we tested the therapeutic efficacy of sCD95Fc in the adjuvant model. As depicted in Figure 8A, left panel, the local recurrent tumor growth was significantly inhibited by the intra-peritoneal application of 25 μg/g-bwt sCD95Fc with the median tumor volume dropping from 942 mm^3^ to 324 mm^3^. This tumor growth inhibition was paralleled by a significant (24%) reduction of the Ki-67 proliferation index (Figure 8A, right panel), showing a major impact of CD95-related inflammatory mechanisms stimulating human pancreatic cancer cell proliferation under in vivo conditions. Moreover, the number of macroscopically visible liver and spleen metastases decreased upon treatment without reaching robust significance.

To further strengthen the relevance of our comprehensive in vitro data under in vivo conditions, a panel of tumor-promoting mRNAs (IL8, IL6, CD44, CD133) in local recurrent tumors were tested after RNA extraction using qRT-PCR as the read-out. As shown in Figure 8B, IL8 and IL6 mRNA levels are decreasing to 37% and 64% compared to controls, respectively, after sCD95Fc application. When the putative stem cell markers CD44 and CD133 (71.5%) were analyzed [41,42,43], this inhibitory effect was also observed for CD133 decreasing to 71.5% compared to the control; however, CD44 did not show any inhibition (Figure 8B lower panel). Finally, the CD95L-blocking effect of sCD95Fc also became visible under in vivo conditions using in situ staining shown with three representative examples in Figure 8C. Interestingly, the CD95 staining revealed a strong immunoreactivity in the stroma of treated mice (upper panels). Since the affinity-purified antiserum was specific for human CD95, this staining pattern indicates the localization of the inhibitory compound. In the three lower panels, a slight reduction of CD95L staining reflects the activity of the inhibitory compound. Yet, this antiserum detects both, human and murine CD95 ligand.

## 4. Discussion

Studies in the past several years have established that the cytokine CD95L member of the TNF superfamily, including TNF-α and TRAIL, plays a crucial role in the progression of tumor growth and metastatic spread through increased production of PIC and CSC (phenotypes) markers as well as activation of NFκB in PDAC [19,21,22,23,44,45]. Palliative chemotherapy has been considered as one of the most effective treatment strategies for advanced PDAC as about 80% of the patients are not permitted to surgical resection [46]. Gemcitabine still remains one of the most widely accepted drugs for the treatment of PDAC [47]. However, PDAC cells have acquired resistance towards gemcitabine-induced apoptosis and exhibit various adverse effects leading to cell survival and metastasis [48]. The impact of the CD95/CD95L system on the mechanism of gemcitabine resistance under the influence of pro-inflammatory responses of death ligands (TNF-α, TRAIL and CD95L) has been not yet appropriately understood.

In the present study, we indicate how the CD95/CD95L system (Figure 1) is involved in the resistance mechanism of gemcitabine under the influence of inflammatory responses induced from TNF-α and TRAIL in the PDAC cells. Our results establish that the up-regulation of CD95 and CD95L upon the treatment of either gemcitabine or death ligands (TNF-α and TRAIL) potentiate the process of chemoresistance, possibly due to the enhanced production of PICs as well as an increase in the expression of CSC marker (stem cell phenotype) in PDAC. Moreover, the blockade of CD95L not only suppresses the gemcitabine-mediated tumor-promoting functions but also sensitized PDAC cells towards gemcitabine-induced cell death by diminishing the pro-inflammatory signaling of TNF-α and TRAIL in a bi- or tri-lateral crosstalk manner (Figure 2, Figure 3 and Figure 5, Figure 6 and Figure 7).

In agreement with previous findings, in our experimental system, we found that gemcitabine not only enhances the expression of PIC and CSC markers but also the mRNA and surface expression of CD95 and CD95L in a dose-dependent manner in vitro (Figure 2A–C) as well as in experimental in vivo conditions (Figure 8). This finding could support that gemcitabine not only selects the resistant cells but also exerts some differentiating effects on tumor cells in the transformation of highly metastatic and stem cell phenotype.

In addition, inhibition of CD95L reduces the expression of PICs, TAPs as well as CSC markers in the dose-dependent manner of PDAC cells in vitro. Moreover, Arora et al. showed that gemcitabine-treated PDAC cells exert increased migratory as well as invasive properties towards a CXCL12 gradient [49]. In our in vitro proliferation assay, we noted that PDAC cells are highly resistant towards gemcitabine-induced cell death. To further investigate the gemcitabine-resistance under the influence of the CD95/CD95L system, an activator of CXCL12 and NFκB, we employed pre-treatment of PDAC cells with potent CD95L inhibitor sCD95Fc prior to gemcitabine treatment. We found that inhibition of CD95L exerts an impact on the sensitization of PDAC cells towards gemcitabine treatment under in vitro conditions, only within a certain dose.

Many recent studies have described an emerged role of these death ligands (CD95L, TNF-α and TRAIL) in the enhanced production of various PICs; chemokines as well as TAPs that are linked with short survival and advanced tumor stages in various malignancies, including PDAC in an NFκB-dependent manner [22,27]. In a recent study, we showed the expression levels of these three death ligands, their cognate receptors in tumor tissues and the correlation of their expression with survival data obtained from 41 PDAC patients by IHC [50].

Since the activation of NFκB plays a central role in the mechanism of chemo-resistance, here, we assume there might be a significant positive feedback loop of gemcitabine/NFκB/CD95/CD95L as gemcitabine could also enhance the expression of CD95 and CD95L. This principle could be extended for TNF-α and TRAIL in the same manner. However, the role of NFκB-mediated pro-inflammatory mechanism in the interaction of these three major death ligands CD95L, TNF-α and TRAIL still remain unclear. In this article, we examined the functional impact of CD95L inhibition on the release of pro-inflammatory factors induced by TNF-α and TRAIL in two distinct PDAC cell lines PancTuI-luc and A818-4.

We observed that TNF-α, TRAIL and CD95L all significantly induced the expression of IL8, IL6, u-PA, MMP7 and MMP9 in both PancTuI-luc as well as A818-4 cell line (Figure 7A,B and Supplementary Figure S5). Further, to support our assumptions that NFκB plays a key role in the death ligand induced pro-inflammatory responses through the TNF-α/NFκB/CD95L or TRAIL/NFκB/CD95L, we analyzed the effects of TNF-α and/or TRAIL treatment on the expression of CD95 and CD95L. We detected that both TNF-α and TRAIL significantly induced the mRNA expression of CD95 and CD95L in both resistant (PancTuI-luc) and sensitive (A818-4) cell lines in a time-dependent manner (Figure 5). However, only the surface expression of mCD95 was found to be increased but not the mCD95L upon TNF-α and TRAIL stimulation (Figure 6). Thus, these findings indicate that there might be a proteolytic cleavage of TNF-α and/or TRAIL-induced mCD95L by metalloproteinases (MMP2, MMP7, ADAM10) and thus, these cleaved or soluble forms of sCD95L might be linked with increased tumor growth and metastasis in an NFκB-dependent manner [51,52].

To support this assumption along with previous findings, we analyzed the impact of broad-spectrum MMP inhibitors on an additional increase in the surface expression of mCD95 and mCD95L (Supplementary Figure S1). Further, an increase in MMP7, MMP9 and uPA expression was detected upon the treatment with TNF-α, TRAIL and rCD95L and upon CD95L inhibition using sCD95Fc, these effects were reduced in both the PDAC cell lines especially with TNF-α stimulation (Supplementary Figure S5). This increased expression of proteases and their activity could further enhance the secretion of sCD95L, which supports the PDAC progression as discussed previously [27,50,51]. Thus, these enhanced expressions of CD95 and CD95L could further support the mechanism of gemcitabine-resistance by increasing the production of PICs and stem cell phenotype of PDAC. The elevated levels of various cytokines, including TNF-α, IL8 and IL6, have been found in the serum of cancer patients treated with gemcitabine [11]. In this study, we investigated the influence of CD95L inhibition on the pro-inflammatory signaling induced by TNF-α and TRAIL in a bi-lateral manner.

Here, a blockade of pancreatic tumor cell-derived CD95L using pharmacological inhibitor sCD95Fc showed considerable reduction in the expression of tumor-promoting genes, including PICs (Figure 7) and TAPs (Supplementary Figure S5) induced upon TNF-α or TRAIL stimulation in a time and concentration-dependent manner in both PDAC cell lines. However, in some cases, sCD95Fc has shown slightly stimulatory effects upon TRAIL treatment with unknown mechanisms, which needs to be further investigated. Our findings indicate there to be crosstalk amongst the non-apoptotic pathways mediated by TNF-α, TRAIL and CD95L. Therefore, the blockade of CD95L might be a significant approach to overcome the problem of chemo-resistance by diminishing the potential pro-inflammatory crosstalk role of TNF-α and TRAIL in the treatment of inflammation-driven tumor progression of PDAC. As a first step towards translation with a therapeutic perspective, we identified an optimal dose of the inhibitor sCD95Fc to be used in an experimental murine orthotopic xeno-transplant tumor model of human PDAC. Thereafter, the therapeutic effect was demonstrated in an adjuvant setting after tumor resection. The remarkable growth reduction of local recurrent tumor mass—a typical characteristic in the clinic after surgical intervention besides liver and other metastases—which was achieved only by blocking CD95 ligand activity clearly underlines the impact of pro-inflammatory, non-apoptotic signaling of “death-receptors” as also shown by us in the TNF system (23).

**Figure 9:**
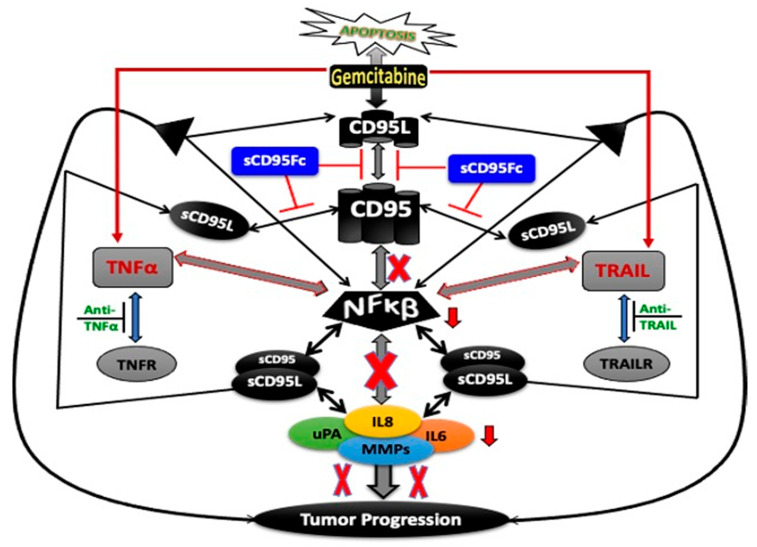
Schematic representation of the mechanism of the interaction amongst TNF-α, TRAIL and CD95L and its impact on chemotherapy. It is the simple chart showing these three signaling cascades crossing over each other’s functions through sharing a common transcription factor NFκB and subsequently inducing expression and secretion of PICs as well as TAPs. Further, it shows how blocking of the CD95L may alter the production of PICs and inflammation derived tumor progression through multiple signals initiated by TNF-α or TRAIL and thus improve the chemotherapeutic effects of gemcitabine.

Together, gemcitabine not only enhanced the expression and activation of death ligands (CD95L and TNF-α) and the activity of survival mediators (NFκB) in a positive feedback loop but also stimulated the production of PICs, TAPs and the enhanced stem cell phenotype of PDAC cells. This entire phenomenon could provide chemo-resistance as well as potentiate the tumor formation and metastasis in PDAC [53,54]. Thus, these findings indicate the importance of the biological effects of the CD95/CD95L system on the unexpected and undesired adverse effects of chemotherapy. The heterogeneity of PDAC becomes already visible in our two model systems (Supplementary Table S2). The death ligand response differs clearly between PancTuI-luc and A818-4 cells, but both behave inversely towards the gemcitabine challenge. A schematic diagram represents a quick review of the entire concept (Figure 9). Thus the blockade of CD95L might provide a potential approach, which not only suppresses the pro-inflammatory responses induced by TNF-α and TRAIL but also enhances the chemotherapeutic effects of gemcitabine by reducing the activation of NFκB and the subsequent production of PICs as well as stem cell phenotype in a combinatorial therapeutic strategy.

## 5. Conclusions

We could demonstrate that gemcitabine stimulates the expression of pro-inflammatory cytokines IL6 and IL8 involving the death receptor CD95 and its ligand CD95L. The CD95L-antagonist sCD95Fc substantially reduced cytokine (TNF-α or TRAIL)-stimulated IL-6 and IL-8 expression and generally lowered the pro-inflammatory status of pancreatic cancer cells as well as their stem cell properties. In a murine xenotransplantation model the antagonist reduced the local recurrent tumor growth after surgical resection of orthotopic primary tumors. Moreover, the unwanted, pro-inflammatory side-effects of gemcitabine were inhibited. Blockade of CD95L might provide a potential therapeutic strategy suppressing pro-inflammatory responses to TNF-α and TRAIL and also enhancing therapeutic effects of gemcitabine.

## Figures and Tables

**Table 1:** Sensitivity of PDAC Cells towards death receptor ligands and Gemcitabine.

Cell Line	TNF-α	TRAIL	CD95L	Gemcitabine
PancTuI-luc	+ +	+ +	−	−
A818-4	−	− −	+ +	+

Symbols: highly resistant, + +; resistant, +; highly sensitive, − −; sensitive −.

## Data Availability

The data presented in this study are available on request from the corresponding author.

## Supplementary Materials

The following are available online at www.mdpi.com/article/10.3390/cancers13215458/s1, Figure S1: Analysis of membrane-bound CD95 and CD95L protein expression after treatment with different MMP inhibitors, Figure S2: Effects of Gemcitabine on the expression of PICs (IL8 and IL6) and PCSC markers (CD44 and CD133) in experimental tumors, Figure S3: Inhibitory Effects of sCD95Fc on the Gemcitabine-induced expression of CD95 and CD95L in pancreatic tumor cells, Figure S4: Pancreatic tumor volume after cell inoculation in a palliative therapy setting, Figure S5: Inhibitory effects of sCD95Fc on uPA, MMP7 and MMP9 mRNA and protein expression in pancreatic cancer cell lines upon TNFα, TRAIL and rCD95L treatment, Human PDAC cell line PancTuI-luc was seeded with 2.8 × 10^5^ cells/well in a 6-well plate, Table S1: List of the PerfectProbe primers used in qPCR, Table S2: Schematic overview on our findings using two distinct pancreatic tumor cell lines PancTuI-luc and A818-4 as model cell lines obtained from two different sources such as primary tumor and ascites respectively.

## References

[1] McGuigan A., Kelly P., Turkington R.C., Jones C., Coleman H.G., McCain R.S. (2018). Pancreatic cancer: A review of clinical diagnosis, epidemiology, treatment and outcomes. World J. Gastroenterol..

[2] Ling Q., Kalthoff H. (2020). Transportome Malfunctions and the Hallmarks of Pancreatic Cancer. Reviews of Physiology, Biochemistry and Pharmacology.

[3] Ellenrieder V., Konig A., Seufferlein T. (2016). Current Standard and Future Perspectives in First- and Second-Line Treatment of Metastatic Pancreatic Adenocarcinoma. Digestion.

[4] Binenbaum Y., Na’ara S., Gil Z. (2015). Gemcitabine resistance in pancreatic ductal adenocarcinoma. Drug Resist. Updates.

[5] Fujiwara Y., Shiba H., Iwase R., Haruki K., Furukawa K., Uwagawa T., Misawa T., Ohashi T., Yanaga K. (2013). Inhibition of nuclear factor kappa-B enhances the antitumor effect of combination treatment with tumor necrosis factor-alpha gene therapy and gemcitabine for pancreatic cancer in mice. J. Am. Coll. Surg..

[6] Harwood F.G., Kasibhatla S., Petak I., Vernes R., Green D.R., Houghton J.A. (2000). Regulation of FasL by NF-kappaB and AP-1 in Fas-dependent thymineless death of human colon carcinoma cells. J. Biol. Chem..

[7] Friesen C., Fulda S., Debatin K.M. (1999). Cytotoxic drugs and the CD95 pathway. Leukemia.

[8] Quint K., Tonigold M., Di Fazio P., Montalbano R., Lingelbach S., Ruckert F., Alinger B., Ocker M., Neureiter D. (2012). Pancreatic cancer cells surviving gemcitabine treatment express markers of stem cell differentiation and epithelial-mesenchymal transition. Int. J. Oncol..

[9] Song Y., Baba T., Li Y.Y., Furukawa K., Tanabe Y., Matsugo S., Sasaki S., Mukaida N. (2015). Gemcitabine-induced CXCL8 expression counteracts its actions by inducing tumor neovascularization. Biochem. Biophys. Res. Commun..

[10] Wang L., Zhang Y., Wang W., Zhu Y., Chen Y., Tian B. (2017). Gemcitabine treatment induces endoplasmic reticular (ER) stress and subsequently upregulates urokinase plasminogen activator (uPA) to block mitochondrial-dependent apoptosis in Panc-1 cancer stem-like cells (CSCs). PLoS ONE.

[11] Rübe C.E., Wilfert F., Uthe D., König J., Liu L., Schuck A., Willich N., Remberger K., Rübe C. (2004). Increased expression of pro-inflammatory cytokines as a cause of lung toxicity after combined treatment with gemcitabine and thoracic irradiation. Radiother. Oncol..

[12] Adamska A., Elaskalani O., Emmanouilidi A., Kim M., Abdol Razak N.B., Metharom P., Falasca M. (2018). Molecular and cellular mechanisms of chemoresistance in pancreatic cancer. Adv. Biol. Regul..

[13] Quinonero F., Mesas C., Doello K., Cabeza L., Perazzoli G., Jimenez-Luna C., Rama A.R., Melguizo C., Prados J. (2019). The challenge of drug resistance in pancreatic ductal adenocarcinoma: A current overview. Cancer Biol. Med..

[14] Trauth B.C., Klas C., Peters A.M., Matzku S., Moller P., Falk W., Debatin K.M., Krammer P.H. (1989). Monoclonal antibody-mediated tumor regression by induction of apoptosis. Science.

[15] Choi C., Xu X., Oh J.W., Lee S.J., Gillespie G.Y., Park H., Jo H., Benveniste E.N. (2001). Fas-induced expression of chemokines in human glioma cells: Involvement of extracellular signal-regulated kinase 1/2 and p38 mitogen-activated protein kinase. Cancer Res..

[16] Desbarats J., Birge R.B., Mimouni-Rongy M., Weinstein D.E., Palerme J.-S., Newell M.K. (2003). Fas engagement induces neurite growth through ERK activation and p35 upregulation. Nat. Cell Biol..

[17] Kleber S., Sancho-Martinez I., Wiestler B., Beisel A., Gieffers C., Hill O., Thiemann M., Mueller W., Sykora J., Kuhn A. (2008). Yes and PI3K bind CD95 to signal invasion of glioblastoma. Cancer Cell.

[18] Ponton A., Clement M.V., Stamenkovic I. (1996). The CD95 (APO-1/Fas) receptor activates NF-kappaB independently of its cytotoxic function. J. Biol. Chem..

[19] Steller E.J., Ritsma L., Raats D.A., Hoogwater F.J., Emmink B.L., Govaert K.M., Laoukili J., Rinkes I.H., van Rheenen J., Kranenburg O. (2011). The death receptor CD95 activates the cofilin pathway to stimulate tumour cell invasion. EMBO Rep..

[20] Toyoshima F., Moriguchi T., Nishida E. (1997). Fas induces cytoplasmic apoptotic responses and activation of the MKK7-JNK/SAPK and MKK6-p38 pathways independent of CPP32-like proteases. J. Cell Biol..

[21] Trauzold A., Roder C., Sipos B., Karsten K., Arlt A., Jiang P., Martin-Subero J.I., Siegmund D., Muerkoster S., Pagerols-Raluy L. (2005). CD95 and TRAF2 promote invasiveness of pancreatic cancer cells. FASEB J..

[22] Ceppi P., Hadji A., Kohlhapp F.J., Pattanayak A., Hau A., Liu X., Liu H., Murmann A.E., Peter M.E. (2014). CD95 and CD95L promote and protect cancer stem cells. Nat. Commun..

[23] Egberts J.H., Cloosters V., Noack A., Schniewind B., Thon L., Klose S., Kettler B., von Forstner C., Kneitz C., Tepel J. (2008). Anti-tumor necrosis factor therapy inhibits pancreatic tumor growth and metastasis. Cancer Res..

[24] Ishimura N., Isomoto H., Bronk S.F., Gores G.J. (2006). Trail induces cell migration and invasion in apoptosis-resistant cholangiocarcinoma cells. Am. J. Physiol. Gastrointest. Liver Physiol..

[25] Leibovich S.J., Polverini P.J., Shepard H.M., Wiseman D.M., Shively V., Nuseir N. (1987). Macrophage-induced angiogenesis is mediated by tumour necrosis factor-α. Nature.

[26] Trauzold A., Siegmund D., Schniewind B., Sipos B., Egberts J., Zorenkov D., Emme D., Roder C., Kalthoff H., Wajant H. (2006). TRAIL promotes metastasis of human pancreatic ductal adenocarcinoma. Oncogene.

[27] Zhou D.H., Trauzold A., Roder C., Pan G., Zheng C., Kalthoff H. (2008). The potential molecular mechanism of overexpression of uPA, IL-8, MMP-7 and MMP-9 induced by TRAIL in pancreatic cancer cell. Hepatobiliary Pancreat. Dis. Int..

[28] Zhou D.H., Yang L.N., Roder C., Kalthoff H., Trauzold A. (2013). TRAIL-induced expression of uPA and IL-8 strongly enhanced by overexpression of TRAF2 and Bcl-xL in pancreatic ductal adenocarcinoma cells. Hepatobiliary Pancreat. Dis. Int..

[29] Pacifico F., Leonardi A. (2006). NF-kappaB in solid tumors. Biochem. Pharm..

[30] Rayet B., Gelinas C. (1999). Aberrant rel/nfkb genes and activity in human cancer. Oncogene.

[31] Karin M., Lin A. (2002). NF-κB at the crossroads of life and death. Nat. Immunol..

[32] RM P.E.P., Stein I., Bramovitch R., Amit S., Kasem S., Gutkovich-Pyest E., Urieli-Shoval S., Galun E., Ben-Neriah Y. (2004). NF-kappaB functions as a tumour promoter in inflammation-associated cancer. Nature.

[33] Kalthoff H., Roeder C., Humburg I., Thiele H., Greten H., Schmiegel W. (1991). Modulation of platelet-derived growth factor A-and B-chain/c-sis mRNA by tumor necrosis factor and other agents in adenocarcinoma cells. Oncogene.

[34] Tepel J., Kruse M.-L., March C., Fiedler A., Kapischke M., Ketterer T., Sipos B., Kremer B., Kalthoff H. (2004). Terminally modified oligodeoxynucleotides directed against p53 in an orthotopic xenograft model: A novel adjuvant treatment strategy for pancreatic ductal carcinoma. Pancreas.

[35] Kettler B., Trauzold A., Röder C., Egberts J.-H., Kalthoff H. (2021). Topology impacts TRAIL therapy: Differences in primary cancer growth and liver metastasis between orthotopic and subcutaneous xenotransplants of pancreatic ductal adenocarcinoma cells. Hepatobiliary Pancreat. Dis. Int..

[36] Egberts J.-H., Schniewind B., Pätzold M., Kettler B., Tepel J., Kalthoff H., Trauzold A. (2008). Dexamethasone reduces tumor recurrence and metastasis after pancreatic tumor resection in SCID mice. Cancer Biol. Ther..

[37] Egberts J.-H., Schniewind B., Sipos B., Sipos B., Kalthoff H., Tepel J. (2007). Superiority of extended neoadjuvant chemotherapy with gemcitabine in pancreatic cancer: A comparative analysis in a clinically adapted orthotopic xenotransplantation model in SCID beige mice. Cancer Biol. Ther..

[38] Ungefroren H., Voss M., Jansen M., Roeder C., Henne-Bruns D., Kremer B., Kalthoff H. (1998). Human pancreatic adenocarcinomas express Fas and Fas ligand yet are resistant to Fas-mediated apoptosis. Cancer Res..

[39] Trauzold A., Schmiedel S., Roder C., Tams C., Christgen M., Oestern S., Arlt A., Westphal S., Kapischke M., Ungefroren H. (2003). Multiple and synergistic deregulations of apoptosis-controlling genes in pancreatic carcinoma cells. Br. J. Cancer.

[40] Tepel J., March C., Ketterer T., Kapischke M., Arlt A., Kremer B., Kalthoff H., Kruse M.-L. (2006). A modified random oligonucleotide-based combination therapy for adjuvant treatment of pancreatic ductal adenocarcinoma. Int. J. Oncol..

[41] Olempska M., Eisenach P.A., Ammerpohl O., Ungefroren H., Fandrich F., Kalthoff H. (2007). Detection of tumor stem cell markers in pancreatic carcinoma cell lines. Hepatobiliary Pancreat. Dis. Int..

[42] Hsu C.-P., Lee L.-Y., Hsu J.-T., Hsu Y.-P., Wu Y.-T., Wang S.-Y., Yeh C.-N., Chen T.-C., Hwang T.-L. (2018). CD44 predicts early recurrence in pancreatic cancer patients undergoing radical surgery. In Vivo.

[43] Zhao S., Chen C., Chang K., Karnad A., Jagirdar J., Kumar A.P., Freeman J.W. (2016). CD44 expression level and isoform contributes to pancreatic cancer cell plasticity, invasiveness, and response to therapy. Clin. Cancer Res..

[44] Qadir A.S., Ceppi P., Brockway S., Law C., Mu L., Khodarev N.N., Kim J., Zhao J.C., Putzbach W., Murmann A.E. (2017). CD95/Fas Increases Stemness in Cancer Cells by Inducing a STAT1-Dependent Type I Interferon Response. Cell Rep..

[45] Teodorczyk M., Kleber S., Wollny D., Sefrin J.P., Aykut B., Mateos A., Herhaus P., Sancho-Martinez I., Hill O., Gieffers C. (2015). CD95 promotes metastatic spread via Sck in pancreatic ductal adenocarcinoma. Cell Death Differ..

[46] Bernstorff W.v., Spanjaard R.A., Chan A.K., Lockhart D.C., Sadanaga N., Wood I., Peiper M., Goedegebuure P.S., Eberlein T.J. (1999). Pancreatic cancer cells can evade immune surveillance via nonfunctional Fas (APO-1/CD95) receptors and aberrant expression of functional Fas ligand. Surgery.

[47] Burris H.r., Moore M.J., Andersen J., Green M.R., Rothenberg M.L., Modiano M.R., Christine Cripps M., Portenoy R.K., Storniolo A.M., Tarassoff P. (1997). Improvements in survival and clinical benefit with gemcitabine as first-line therapy for patients with advanced pancreas cancer: A randomized trial. J. Clin. Oncol..

[48] Singh S., Srivastava S., Bhardwaj A., Owen L., Singh A. (2010). CXCL12–CXCR4 signalling axis confers gemcitabine resistance to pancreatic cancer cells: A novel target for therapy. Br. J. Cancer.

[49] Arora S., Bhardwaj A., Singh S., Srivastava S.K., McClellan S., Grizzle W.E., Owen L.B., Singh A.P. (2013). An undesired effect of chemotherapy: Gemcitabine promotes pancreatic cancer cell invasiveness through upregulation of CXCR4. Cancer Res..

[50] Gaertner F., Krüger S., Röder C., Trauzold A., Röcken C., Kalthoff H. (2018). The expression of death receptor systems TRAIL-R1/-R2/-R4, CD95 and TNF-R1 and their cognate ligands in pancreatic ductal adenocarcinoma. Histol. Histopathol..

[51] Almendro Navarro V., Ametller E., Garcia Recio S., Collazo O., Casas I., Augé Fradera J.M., Maurel Santasusana J., Gascón P. (2009). The Role of MMP7 and its cross-talk with the FAS/FASL system during the acquisition of chemoresistance to oxaliplatin. PLoS ONE.

[52] Mitsiades N., Yu W.H., Poulaki V., Tsokos M., Stamenkovic I. (2001). Matrix metalloproteinase-7-mediated cleavage of Fas ligand protects tumor cells from chemotherapeutic drug cytotoxicity. Cancer Res..

[53] Villunger A., Egle A., Kos M., Hartmann B.L., Geley S., Kofler R., Greil R. (1997). Drug-induced apoptosis is associated with enhanced Fas (Apo-1/CD95) ligand expression but occurs independently of Fas (Apo-1/CD95) signaling in human T-acute lymphatic leukemia cells. Cancer Res..

[54] Wesselborg S., Engels I.H., Rossmann E., Los M., Schulze-Osthoff K. (1999). Anticancer drugs induce caspase-8/FLICE activation and apoptosis in the absence of CD95 receptor/ligand interaction. Blood.

