# Supplementary material for: CD95L Inhibition Impacts Gemcitabine-Mediated Effects and Non-Apoptotic Signaling of TNF-α and TRAIL in Pancreatic Tumor Cells
Source: Cancers (Basel). 2021 Oct 30;13(21):5458. doi: 10.3390/cancers13215458 (PMC8582466; doi:10.3390/cancers13215458)
Supplement: Supplementary file 1 [file cancers-13-05458-s001.zip › cancers-1398281-supplementary.pdf]

CD95L Inhibition Impacts Gemcitabine-Mediated Effects and Non-Apoptotic Signaling of TNF-α and TRAIL in Pancreatic Tumor Cells

Khalid Rashid, Christian Röder, Freya Goumas, Jan-Hendrik Egberts and Holger Kalthoff

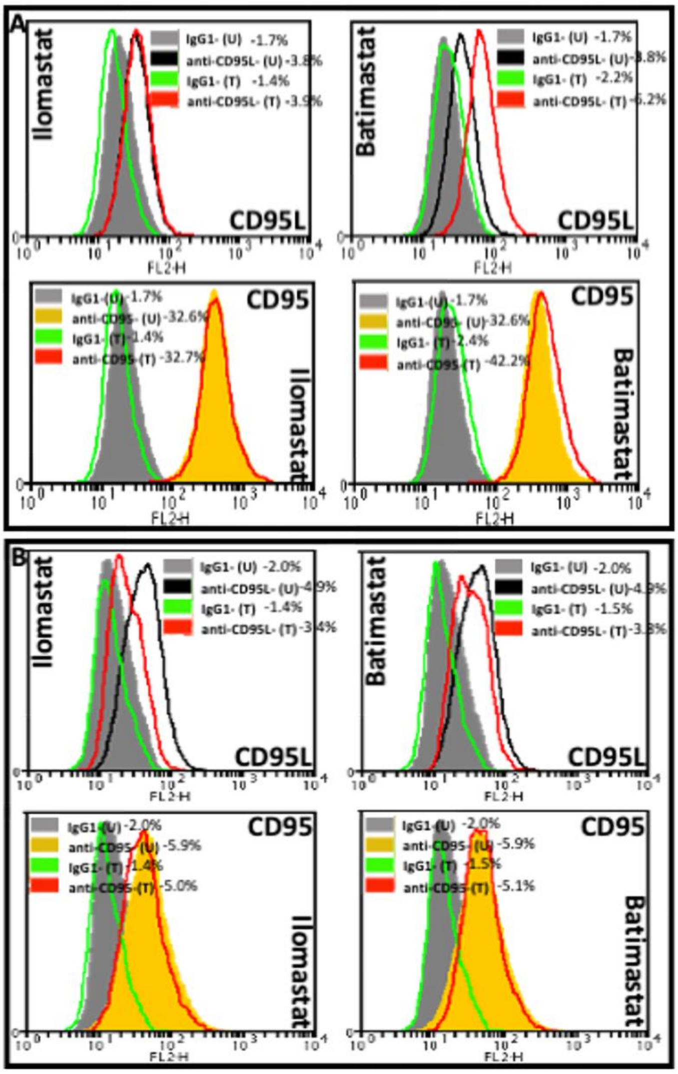

Figure S1. Analysis of membrane-bound CD95 and CD95L protein expression after treatment with different MMP inhibitors. CD95 and CD95L cell surface expression was assayed in (non-permeabilized) PancTuI-luc (A) and A818-4 (B) using phycoerythrin-conjugated mouse anti-human-CD95L antibody NOK-1 (Black line peaks-untreated and Red line peaks -treated with Ilomastat or Batimastat; synthetic and broad-spectrum inhibitors of metalloproteinases) and mouse anti-human-CD95 antibody DX-2 (filled yellow peaks –untreated-U and Red line peaks –treated-T with Ilomastat or Batimastat) and or an irrelevant antibody (IgGl-isotype control) used for the determination of background staining (Grey filled peaks-untreated-U and Green line peaks-treated-T with Ilomastat; left row or Batimastat; right row). Raw data were statistically evaluated for histogram plots from two biological replicates (n=2) as described in "Section 2.

Supplementary Figure-S2, PDAC is one of the most inflammatory and malignant diseases. Due to such conditions, in most of the cases, palliative treatment is the only therapeutic option. Palliative treatment of the patients is achieved by either chemotherapy or

radiotherapy to slow down or stop cancer growth. However, in some cases where surgical resection is possible, an adjuvant therapy can be applied which usually refers to surgical removal of cancerous tissues followed by chemo- or radiotherapy to further decrease the risk of cancer recurrence. Here, we have investigated the effects of Gemcitabine treatment on the pro-inflammatory cytokines (PICs), PCSC markers, as well as CD95L in both palliative and adjuvant settings. Interestingly, we have noted that Gemcitabine induces the expression of PICs and PCSC markers in both the settings, indicating a potential role of Gemcitabine in inflammation and stemness in PDAC. This pro-inflammatory function of gemcitabine affects also CD95L expression and gave us a rational to further investigate the underlying mechanisms involved in Gemcitabine-induced PDAC cell growth and survival.

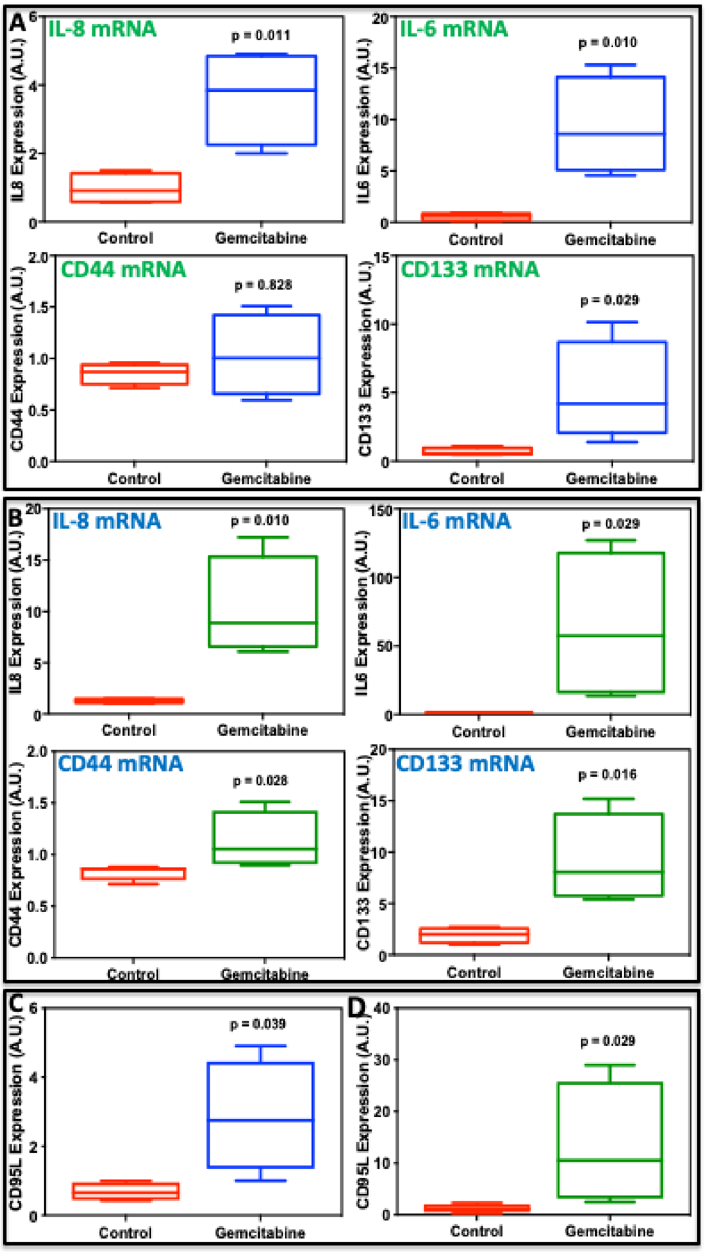

Figure S2. Effects of Gemcitabine on the expression of PICs (IL8 and IL6) and PCSC marker (CD44 and CD133) in experimental tumors. Female SCID beige mice bearing PancTuI-luc tumor xenografts were treated with i.p. injections of Gemcitabine (treated group- 5 µg/g bwt) in the palliative setting (A, C) or adjuvant setting (B, D) along with sodium chloride 0.9% (control group) for the time period

described elsewhere. The tissues were homogenized and lysed in lysis buffer; total RNA was isolated, and assayed for IL8, IL6, CD44, CD133 and CD95L via a PerfectProbe PCR Assay. The resulting values of the corresponding genes were normalized against reference gene UBC and expressed as arbitrary unit (A.U.). Each group contained eight (A) or nine (B) mice and the normal distribution of the data was tested by the Shapiro-Wilk method. A student t-test or Mann-Whitney test was performed to compare the significance (P < 0.05) of each treated group vs. the control group: p values were indicated on the bar of individual gene. Data represent means ± SEM of biological replicates (n=8-9).

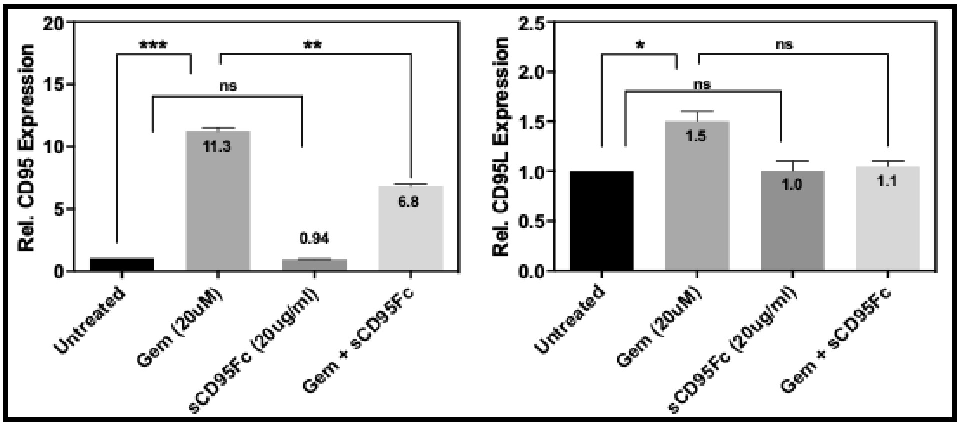

Figure S3. Inhibitory Effects of sCD95Fc on the Gemcitabine-induced expression of CD95 and CD95L in pancreatic tumor cells. Human PDAC cell line PancTuI-luc was seeded with 2.8 x 10^5 cells/well in a 6-well plate. Cells were either treated with 0.9 % saline or untreated as controls or treated with Gemcitabine (20µM) alone or in combination with sCD95Fc (20 µg/ml). Cells were lysed in RNA Lysis Buffer T, total RNA was isolated, and assayed for CD95 (left panel) and CD95L (right panel) via PerfectProbe real-time RT-PCR assays. Relative expression was normalized to the control and reference gene UBC and expressed as arbitrary unit (A.U.). Controls were set as 1 in each experiment. Data represent means ± SD of biological replicates (n=2) with three technical replicates each. In case of statistical significance, asterisks indicate the corresponding significance levels (Student's t-test; *, P < 0.05; **, P < 0.01; ***, P < 0.001; n.s., not significant).

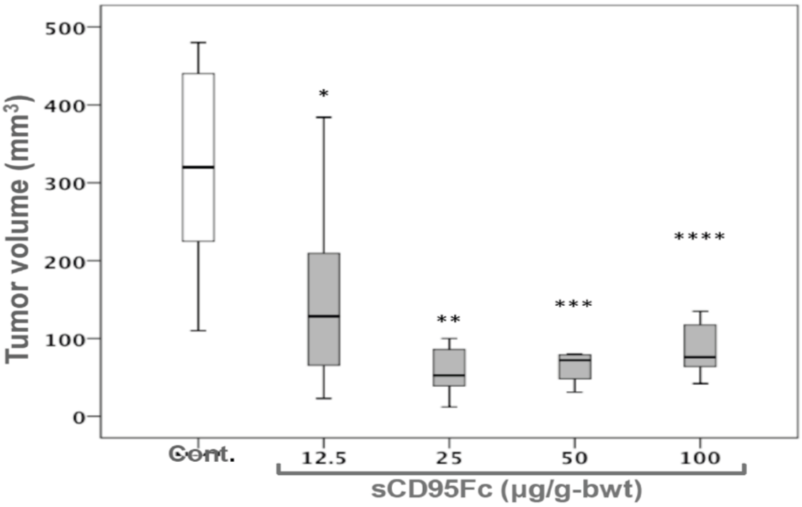

Figure S4. Pancreatic tumor volume after cell inoculation in a palliative therapy setting. Female SCID beige mice (n=8/group) bearing PancTuI-luc tumor xenografts were treated intraperitoneally with either sodium chloride 0.9% (control group) or with different concentrations (12.5,25,50,100 µg/g bwt) of sCD95Fc (treated group). The tumor volume was determined on day 28 after tumor cell inoculation. The normal distribution of the data was tested by the Shapiro-Wilk method and in case of significance, Mann-Whitney U-tests were performed between treated group vs. the control group and the statistical significance levels were indicated as asterisks at the corresponding boxplots (*, P < 0.05; **, P < 0.01; ***, P < 0.001; ****, P < 0.0001).

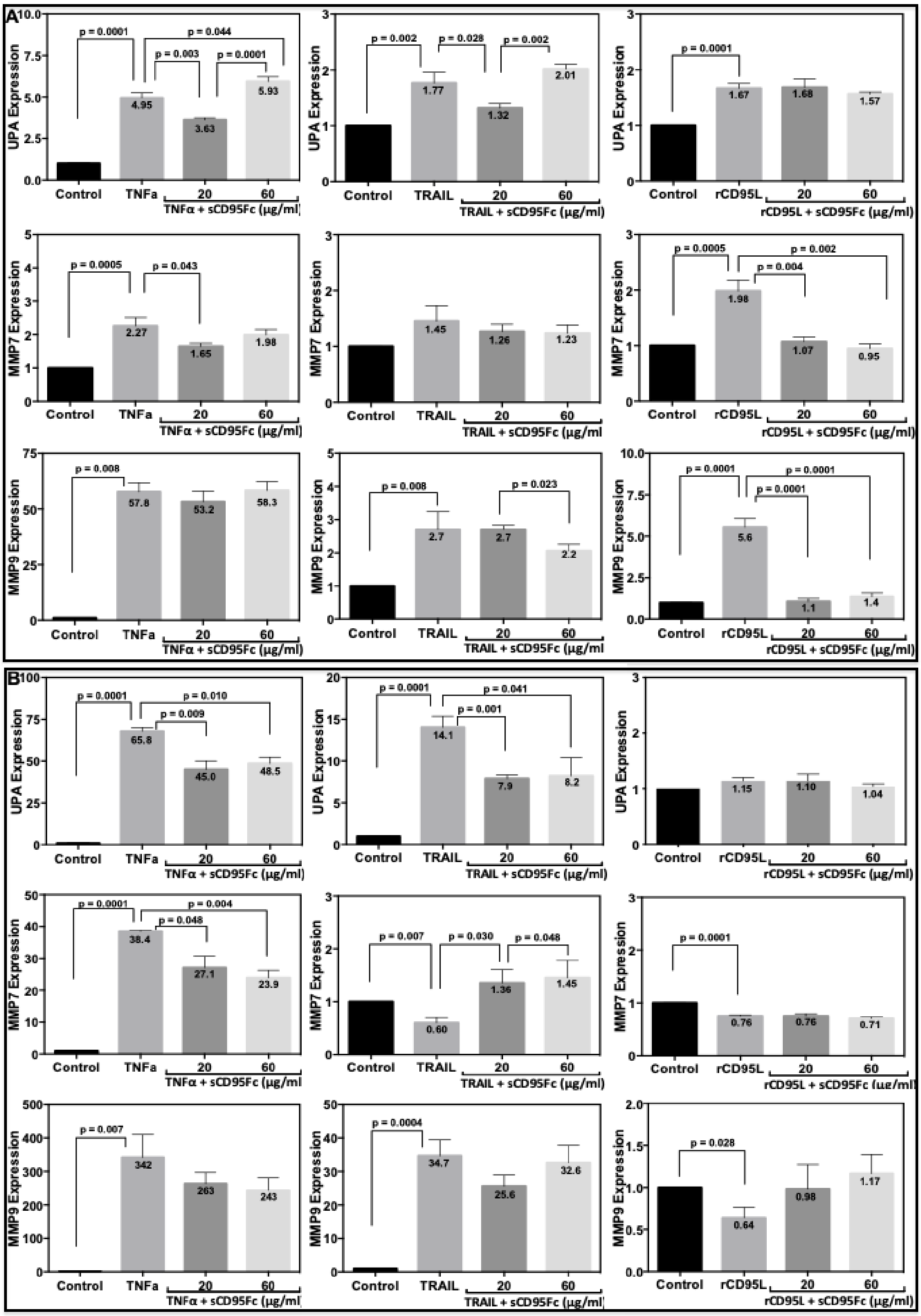

Figure S5. Inhibitory effects of sCD95Fc on uPA, MMP7 and MMP9 mRNA and protein expression in pancreatic cancer cell lines upon TNFα, TRAIL and rCD95L treatment. Human pancreatic cancer cells PancTuI-luc (A) and A818-4 (B) were seeded with 2.8 x 10^5 cells/well in 6-well plates. Cells were either left untreated as control (black bar) or stimulated with 50 ng/ml TNF-α or 50 ng/ml TRAIL or 100 ng/ml rCD95L (grey bar), TNF-α $\pm$ sCD95Fc (20 µg/ml) or TRAIL $\pm$ sCD95Fc (20 µg/ml) or rCD95L $\pm$ sCD95Fc (20 µg/ml) (dark grey) and TNF-α $\pm$ sCD95Fc (60 µg/ml) or TRAIL $\pm$ sCD95Fc (60 µg/ml) or rCD95L $\pm$ sCD95Fc (60 µg/ml) (light grey) for 36 hrs. Cells were lysed in RNA Lysis Buffer T; total RNA was isolated, and assayed for uPA (upper), MMP7 (middle) and MMP9 (lower) by PerfectProbe RT-PCR assays (A and B). Relative expression was normalized to the control and reference genes UBC or RPL13A and expressed as arbitrary unit (A.U.). In case of significance, P-values were indicated. Data represent means ± SEM of biological replicates (n=3).

Table S1. List of the PerfectProbe primers used in qPCR.

Gene	F/R	PerfectProbe Primer sequence (5′-3′)
**CD95**	**F**	TGTAGTATGAATGTAATCAGTGTATGT
	**R**	GATATTTCAGCAAAAGGTCATAGC
**CD95L**	**F**	CTCCTCAACTCACCTAATGTTTATG
	**R**	TTTCATGCTTCTCCCTCTTCAC
**IL8**	**F**	CAGAGACAGCAGAGCACAC
	**R**	AGCTTGGAAGTCATGTTTACAC
**IL6**	**F**	GCAGAAAACAACCTGAACCTT
	**R**	ACCTCAAACTCCAAAAGACCA
**CD44**	**F**	AATGCCTTTGATGGACCAAT
	**R**	TAGGGTTGCTGGGGTAGATG
**CD133**	**F**	AGCTGCTTCCTCAGACATGC
	**R**	GAGTGGCCGGGTTCTAGAGT
**UBC, ATP5B, SDHA & RPL13A**		The UBC (ubiquitin C), ATP5B (ATP synthase subunit beta), SDHA (succinate dehydrogenase) and RPL13A (60s ribosomal protein 13La) gene expression primers are part of the 6-gene human geNorm™ Housekeeping Gene Selection Kit with PerfectProbe, cat# ge-PP-6-hu (PrimerDesign Ltd., Southampton, UK)

Table S2. Schematic overview on our findings using two distinct pancreatic tumor cell lines PancTuI-luc and A818-4 as model cell lines obtained from two different sources such as primary tumor and ascites respectively.

**Responses of Death Ligands and Gemcitabine in PancTuI-luc and A818-4**		
**Sources, Properties and Treatment Response**	**PancTuI-luc**	**A818-4**
**Sources of the two PDAC cell lines**	Primary tumor	Ascites
**CD95 mRNA and Surface Protein expression**	Very high	High (H)
**CD95L mRNA and Surface Protein expression**	Low (L)	High
**Effect on Cell Growth Pattern (Proliferation)**		
**TNF-α response on cell growth pattern**	(H) Resistant	Sensitive
**TRAIL response on cell growth pattern**	(H) Resistant	(H) sensitive
**rCD95L response on cell growth pattern**	Resistant	(H) resistant
**Gemcitabine response on proliferation**	sensitive	Resistant
**sCD95Fc (CD95L Inhibitor) effect on proliferation**	No effect	No effect
**Effect of CD95Fc on TNF-α-induced proliferation**	Neutralizing	Additive
**Effect of CD95Fc on TRAIL-induced proliferation**	Additive	Additive
**Effect of CD95Fc on rCD95L-induced proliferation**	No effect	Neutralizing
**sCD95Fc + Gemcitabine effect on proliferation**	Inhibition	Inhibition
**Effect on Gene Induction/Expression**		
**TNF-α/TRAIL-induced CD95/CD95L expression**	High	Very high
**TNF-α/TRAIL-induced IL8/IL6 expression**	High	Very high
**Effect sCD95Fc on TNF-α-induced CD95/CD95L**	None	Only on CD95L
**Effect sCD95Fc on TRAIL-induced CD95/CD95L**	Only on CD95L	Both
**Effect sCD95Fc on rCD95L-induced CD95/CD95L**	Inhibition	None
**Effect sCD95Fc on TNF-α/TRAIL-induced IL8/IL6**	Low	High
**Effect of sCD95Fc on endogenous CD95/CD95L**	Low/None	Inhibition
**Effect of sCD95Fc on endogenous IL8/IL6**	None	Inhibition
**(H) - High**		

We analyzed the mRNA and surface expression of CD95 and CD95L and both cell lines show expression of both, CD95 and CD95L at the mRNA level as well as surface protein revealing different/inverse expression levels in both cell lines. Further, the impact of exogenous TNF-α or TRAIL or CD95L (signaling) stimuli as well as gemcitabine treatment on cell growth pattern and induction/production of tumor-promoting genes; TPGs

(pro-inflammatory cytokines; PICs- IL8/IL6 and tumor-associated proteases; TAPs-uPA/MMPs) was analyzed in these two cell lines. Very contrasting characteristic features were observed on cell growth pattern and degree of gene induction towards gemcitabine as well as all the three death ligands stimuli depending on the type of cells and the time of stimulus. Furthermore, inhibition of CD95L considerably reduced the expression of these TPGs (PICs/TAPs) induced upon the TNF-α or TRAIL stimulation including the potential neutralization of rCD95L responses in both PDAC cell lines. Intriguingly, the impact of inhibition of endogenous CD95L shows no inhibitory effects in PancTuI-luc while a significant inhibition is reported in case of A818-4.
